# Toxicological and bio-distribution profile of a GM-CSF-expressing, double-targeted, chimeric oncolytic adenovirus ONCOS-102 – Support for clinical studies on advanced cancer treatment

**DOI:** 10.1371/journal.pone.0182715

**Published:** 2017-08-10

**Authors:** Lukasz Kuryk, Lotta Vassilev, Tuuli Ranki, Akseli Hemminki, Aila Karioja-Kallio, Onerva Levälampi, Antti Vuolanto, Vincenzo Cerullo, Sari Pesonen

**Affiliations:** 1 Targovax Oy, Helsinki, Finland; 2 ImmunoViroTherapy lab, Division of Pharmaceutical Biosciences & Centre for Drug Research, Faculty of Pharmacy, University of Helsinki, Helsinki, Finland; 3 Department of Virology, National Institute of Public Health–National Institute of Hygiene, Warsaw, Poland; 4 University of Helsinki, Faculty of Medicine, Department of Pathology, Cancer Gene Therapy Group, Helsinki, Finland; 5 Department of Oncology, HUCH, Helsinki, Finland; 6 TILT Biotherapeutics Ltd., Helsinki, Finland; 7 Faculty of Medicine, University of Helsinki, Helsinki, Finland; Medical University Innsbruck, AUSTRIA

## Abstract

The purpose of this work was to carry out preclinical toxicity and bio-distribution studies required for regulatory approval of a clinical trial application for Phase I clinical studies of ONCOS-102 (Ad5/3-D24-GM-CSF) for therapy of advanced cancers (NCT01598129). The study design, route of administration and dosage differs from the clinical protocol and in more detail, investigate bio-distribution and toxicological profile of ONCOS-102 treatment in animal model. The study was carried out in 300 hamsters divided into nine test groups–three bio-distribution groups and six groups for analysis of toxicity. Hamsters received ONCOS-102 by intracardial, intraperitoneal or subcutaneous injections. Additionally, one group was administered twice a week with intraperitoneal injections of Cyclophosphamide. The control animals were administered with NaCl solution without ONCOS-102 in the same volume and the same way. No adverse effects of repeated administration of ONCOS-102 including body weight, food consumption, hematology and clinical chemistry parameters, histopathology and bio-accumulation were observed in the course of 6-month administration and following 3- month recovery period. All obtained findings indicate the treatment clinically safe.

## Introduction

Advanced, metastatic tumors often exhibit resistance to standard therapies, and thus novel, safer and more effective treatment modalities are in high need [1, 2].

Oncolytic adenoviruses are promising agents in cancer therapy. Their efficacy and safety has been proven in several clinical studies [3–5].

ONCOS-102 is a serotype 5 human adenovirus with a chimeric 5/3 capsid for enhanced cancer cell transduction due to alternative receptor usage, and a 24 bp deletion in the Rb binding site of the E1A gene, rendering the viral replication to target cells [3]. Importantly, ONCOS-102 codes for human granulocyte macrophage colony-stimulating factor (GM-CSF), a potent immunostimulatory cytokine for enhanced anti-tumor immunity by recruiting and activating APC (antigen presenting cells) [6, 7].

ONCOS-102 induces systemic anti-tumor T cell response in cancer patients by directly lysing cancer cells and concomitantly causing a modulation of tumor microenvironment from Th2- to Th1-type [8]. Next ONCOS-102 sensitizes tumor cells to other immunotherapies by inducing a T-cell positive phenotype to an initially T-cell negative tumor mass [8]. Importantly presented double mode of action of ONCOS-102 is an important factor needed to overcome the major obstacle with regards to cancer immunotherapy—suppressive tumor microenvironment [1].

The aim of the study was to evaluate the toxicological profile and bio-distribution of ONCOS-102 after intracardial and repeated intraperitoneal or subcutaneous administration in Syrian hamsters. The study was carried out in order to support clinical trial application of ONCOS-102 (NCT01598129) for therapy of advanced cancers. The study was successfully accomplished: toxicological and bio-distribution results and conclusions are presented in this paper.

## Materials and methods

### GLP compliance

This pre-clinical study was performed in compliance with the Organization for Economic Co-operation and Development (OECD), Principles of Good Laboratory Practice (GLP) C (97)186/Final, Directive 2004/10/EC, The Czech law No. 378/2007 and Decree of Ministry of Health and Ministry of Agriculture of the Czech Republic No. 86/2008 of Collection of laws about Good Laboratory Practice for testing of drugs.

### Animal welfare act compliance, ethics statement

The study was prepared for this type of experiment and approved by the Institutional Animal Care and Use Committee (IACUC) and the Committee for Animal Protection of the Ministry of Industry and Trade of the Czech Republic (10/2009). Animal care was in compliance with the SOPs of BioTest s.r.o., the European convention for the protection of vertebrate animals used for experimental and other scientific purposes (ETSV123), the Czech collection of laws No. 246/1992, inclusive of the amendments, on the Protection of animals against cruelty, and Public Notice of the Ministry of Agriculture of the Czech Republic, Collection of laws No. 207/2004 as amended, on keeping and exploitation of experimental animals. BioTest s.r.o., is a holder of the accreditation Certificate for user's issued by Central Committee for Animal Protection of the Czech republic.

Procedures used in this report are designed to conform to accepted practices and to minimize or avoid causing pain, distress, or discomfort to the animals. The number of animals selected for use in this study was considered to be the minimum number necessary to meet scientific and regulatory guidelines for this type of study. Twelve animals did not survive till their scheduled necropsy. One animal (D3-TOX group) died during the acclimation period before the start of the treatment. One animal from D2-BIO and three animals from D2-TOX died on Day 1 during intracardial needle insertion, but before the virus injection. One animal from group C-TOX, one animal from D2-TOX, and one animal from D3-TOX died on Day 1 within several minutes after the intracardial virus administration. Four animals, which died directly during administration, but before virus injection, were not necropsied, other three, which died within several minutes after the administration, were necropsied. The cause of death of all these animals was related to the unsuccessful intracardial application and was not related to the treatment. One animal from D2-TOX was found dead on Day 27 (after 4 administrations) and one animal from D1-TOX died on Day 104. The cause of the death was circulatory failure. One male from group D3-TOX was euthanized in the 18th week of the study (Day 120) due to loss of incisors related to high age. One male from group D3-TOX died in ether narcosis during final blood sampling (Day 255). All other animals survived till their scheduled necropsy.

### Test item–ONCOS-102

Adenovirus ONCOS-102 is a class II genetically modified micro-organism (GMM). The engineering of ONCOS-102 has been described previously [3]. ONCOS-102 was produced by Biovian (Turku, Finland) according to GLP and stored at -80°C until use.

### Test system–syrian hamsters

The study was carried out with 300 hamsters (Syrian hamsters, *Mesocricetus auratus*, males and females, supplier by AnLab s.r.o, Czech Republic.) divided into nine test groups–three groups for bio-distribution (C-BIO, D2-BIO and D2-BIO SC) and six groups for toxicity analysis (C-TOX, D1-TOX, D2-TOX, D2-TOX SC, D2-TOX CP, D3-TOX). Animals were sorted according to the body weight, and allocated to the dose group. Hamsters were housed individually in Macrolon 2000P cages (Tecniplast, Italy) in barrier laboratory conditions (BSL-2). Room temperature was 20–24°C and the relative humidity between 30–70%. Fluorescent lighting provided illumination approximately 12 hours per day. Feed, water containers and bedding were changed and sanitized at least once weekly. Hamsters were acclimated for 7 days.

### Dose procedure

Each hamster in a dose group (except D2-TOX SC and D2-BIO SC) received the indicated dose (Table 1) of ONCOS-102 in sterile saline solution by intracardial injection (in isoflurane anaesthesy) on day 1 and intraperitoneal injections on days 4, 8, and 15. D2-TOX SC and D2-BIO SC groups were administered in the same intervals subcutaneously only. Additionally, group D2-TOX CP was administered twice a week with intraperitoneal injections of Cyclophosphamide (120M1253V, Sigma Aldrich, Germany) in dose of 20 mg/kg (diluted in saline). Control animals were administered with saline solution without ONCOS-102 in the same volume and the same way. Administration followed in the same manner on days 29, 57, 85, 113, 141 and 169. The first day of dosing is designated as day 1 of the study.

**Table 1:** Treatment groups for toxicological and bio-distribution studies–allocation and dosing.

Group Designation	Dose	No. of animals	
		males	females
C-BIO(Control)	0 (i.c./i.p.)	15	15
D2-BIO (Middle Dose)	4.5 x 10^10^ v.p./kg (i.c./i.p.)	15	15
D2-BIO SC (Middle Dose)	4.5 x 10^10^ v.p./kg (s.c.)	5	5
C-TOX (Control)	0 (i.c./i.p.)	25	25
D1-TOX (Low Dose)	4.5 x 10^9^ v.p./kg (i.c./i.p.)	20	20
D2-TOX (Middle Dose)	4.5 x 10^10^ v.p./kg (i.c./i.p.)	20	20
D2-TOX CP (Middle Dose)	4.5 x 10^10^ v.p./kg (i.c./i.p.); 20mg/kg (i.p.)	20	20
D2-TOX SC (Middle Dose)	4.5 x 10^10^ v.p./kg (s.c.)	5	5
D3-TOX (High Dose)	4.5 x 10^11^ v.p./kg (i.c./i.p.)	25	25

BIO–bio-distribution groups, TOX–toxicity groups

ONCOS-102—Test item, Cyclophosphamide/CP–additive item

v.p./kg—virus particles per kg

(i.c./i.p.)–intracardial/intraperitoneal administration, (s.c./SC)–subcutaneous administration

The study design, dosage and route of administration in the study was consulted and agreed with regulatory authorities: European Medicines Agency (EMA) and Food and Drug Administration (FDA). Medium (4.5 x 10^10^ VP/kg), high (4.5 x 10^11^ VP/kg), and very high (4.5 x 10^12^ VP/kg) doses of ONCOS-102 (i.e. 10x to 1000x the intended clinical dose w/w) were given with CPO (20 mg/kg i.p.) on Day 1 (by intracardial [i.c.] injection), and Days 4, 8 and 15 (by i.p. injection).

### Clinical observations

#### Daily observations

All hamsters were observed daily for clinical signs, morbidity or mortality during acclimation and administration period and additionally 1 hour after each ONCOS-102 administration. Onset, duration and severity of any signs were recorded.

#### Body weight and food consumption

All hamsters were individually weighed at delivery, before the first administration, then weekly during the administration and recovery period and before the necropsy. Individual food consumption was recorded weekly during acclimation, administration and recovery periods in all TOX groups only.

### Sampling and analysis

#### Blood analyses

Blood samples for hematology (S1 Table) and clinical chemistry (S2 Table), and for neutralizing antibodies, were collected from all TOX animals before administration (day 7), at days 29, 190 and 255, and at days 29, 190 and 255, respectively. The animals were fasted for approximately 12–18 hours before blood sampling, but water was provided ad libitum. Blood samples were drawn under ether anesthesia from the retro-orbital venous.

#### DNA isolation and qPCR

The number of copies of adenoviral sequence (E1 region) and hamster Gapdh sequence were determined in DNA samples. Samples of feces were isolated using NucleoSpin kit, samples of urine and buccal swabs were isolated using NucleoSpin Blood DNA isolation and serum samples were isolated using NucleoSpin Blood DNA isolation kit according to the manufacturer´s instructions (Macherey-Nagel, Germany). In tissue samples the concentration of adenoviral sequence (primer FWE1: 5´-TCC GGT TTC TAT GCC AAA CCT-3´, primer RVE1: 5´- TCC TCC GGT GAT AAT GAC AAG A-3´, probe adenoE1: 5- ATC GAT CCA CCC AGT GAC GAC-3) was normalized by the concentration of hamster’s Gapdh sequence (primer FWGapdh: 5´- CAC CGA GGA CCA GGT TGTC T-3´, primer RVGapdh: 5´-CAT ACC AGG AGA TGA GCT TTA CGA-3´, probe Gapdh: 5-CAA TGC CAG CCC CAG CATC A-3) or by the total amount of DNA. Tissues and other samples (brain, heart, optic nerves, liver, lungs, spleen, kidney, gonads, bone marrow, buccal swab, urine, feces) for analysis of bio-distribution were collected on day 3 in groups C-BIO and D2-BIO and on day 29 in all BIO groups and on day 183 in groups C-BIO and D2- BIO.

#### Analysis of ONCOS-102-specific neutralizing antibodies

Serum was snap-frozen upon collection and stored below -70°C until transportation to analysis. Neutralizing antibody measurements were done as previously described [9].

### Necropsy

Necropsy was performed in TOX groups on day 29, 190 and 256. All animals were weighed and examined externally. Any abnormalities were recorded with details of location, color, shape and size. Samples of all tissues for analysis of neutralizing antibodies were taken in parallel with samples collected for histology.

Whole organs or samples of the collected tissues were preserved in 4% neutral buffered formaldehyde (S3 Table). The eyes, optic nerves, testes and epididymides were fixed in Davidson's fluid and then moved to 4% neutral buffered formaldehyde.

Histopathology was performed for organs and tissues listed in S3 Table. Histopathological examination was performed in all TOX animals except for D2-TOX SC (middle dose) group. Tissues from all animals were paraffin embedded, cut at a nominal thickness of approximately 5μm, stained with haematoxylin and eosin (HE) and examined microscopically.

### Data compilation and statistical analysis

Group mean, medians and standard deviations were calculated for body weights, food consumption, hematology, clinical chemistry and DNA isolation using Graph Pad Prism software, v4 (GraphPad Software, San Diego, CA). In addition, values were subjected to analysis of variance (ANOVA) followed by Dunnett’s Test. Additional statistical analyses were performed by Kruskal-Wallis test.

## Results

### Clinical observations

Twelve animals did not survive till their scheduled necropsy. The cause of death of all these animals was related to the unsuccessful intracardial application and was not related to the treatment agent. All tested animals were in good health status, and no severe signs of toxicity were observed in-group. Blindness of right eye was observed in one male from the D2-TOX group, probably as a consequence of blood sampling from retro-orbital plexus without relation to the treatment. Tumescent testes were repeatedly observed in three animals from the same group and in one male from group D1-TOX. All the other animals showed no clinical signs of toxicity observed as: changes in activity, response to handling or other recorded signs.

#### Body weight

**Fig 1:**
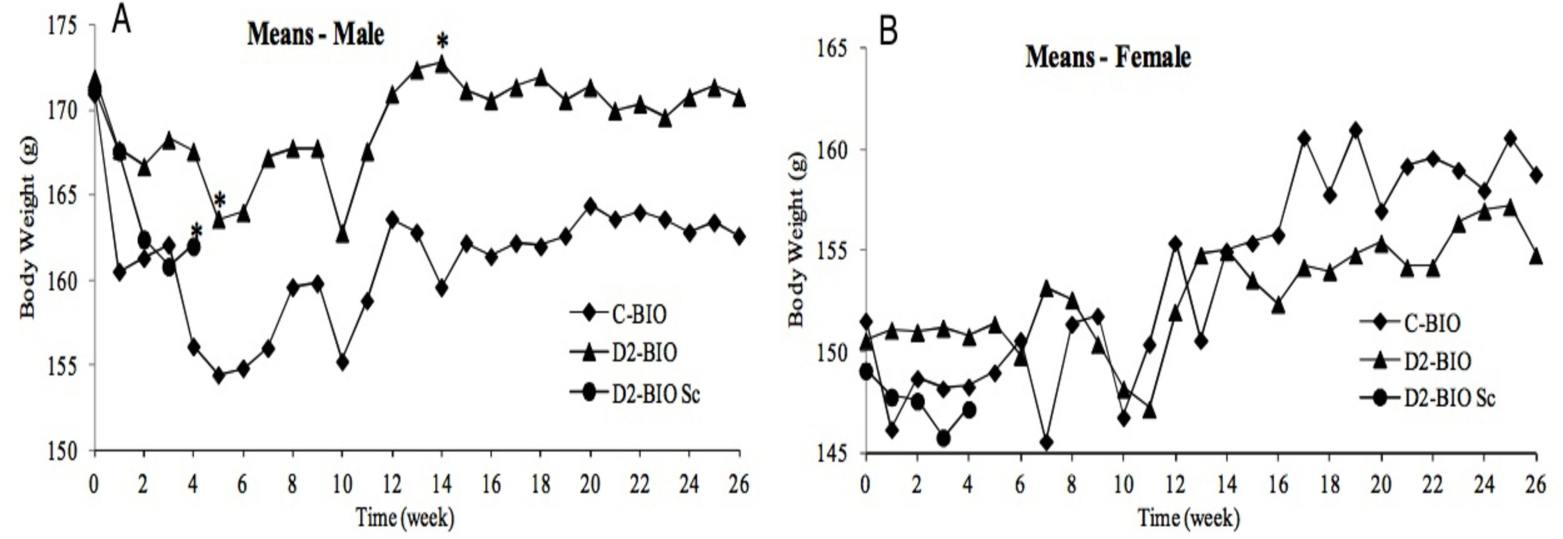
Body weight (g)–BIO groups. (A)–males. (B)–females.

**Fig 2:**
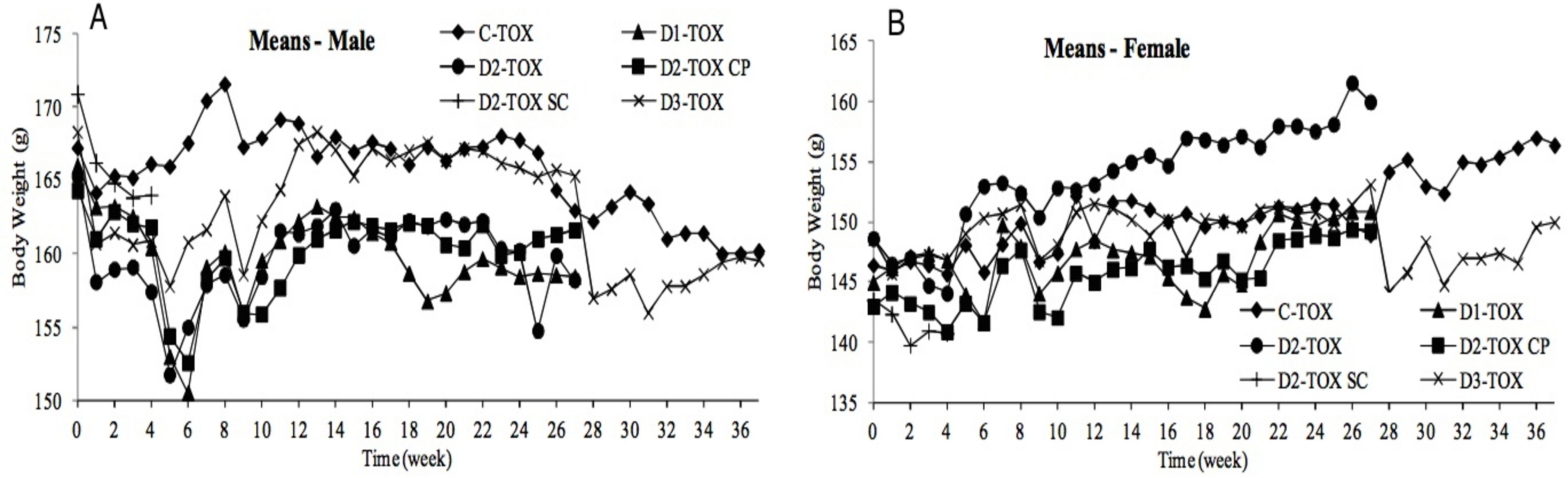
Body weight (g)–TOX groups. (A)–males. (B)–females.

The mean body weight of all hamster groups decreased after the first administration when compared to the baseline, probably due to excessive handling (Figs 1 and 2). The average body weight in the course of the acclimation was expressed as baseline. In the first 6 weeks of administration period mean body weight of males in all treated groups slightly decreased, but subsequently returned to normal values. In the course of administration and recovery periods, the mean body weight was virtually constant or slightly varied in both sexes of all groups, which was in accordance with the age of the animals. No statistically significant decrease in the mean body weight was observed in any test group when compared to the control. No influence of ONCOS-102 on body weight was observed.

#### Food consumption

**Fig 3:**
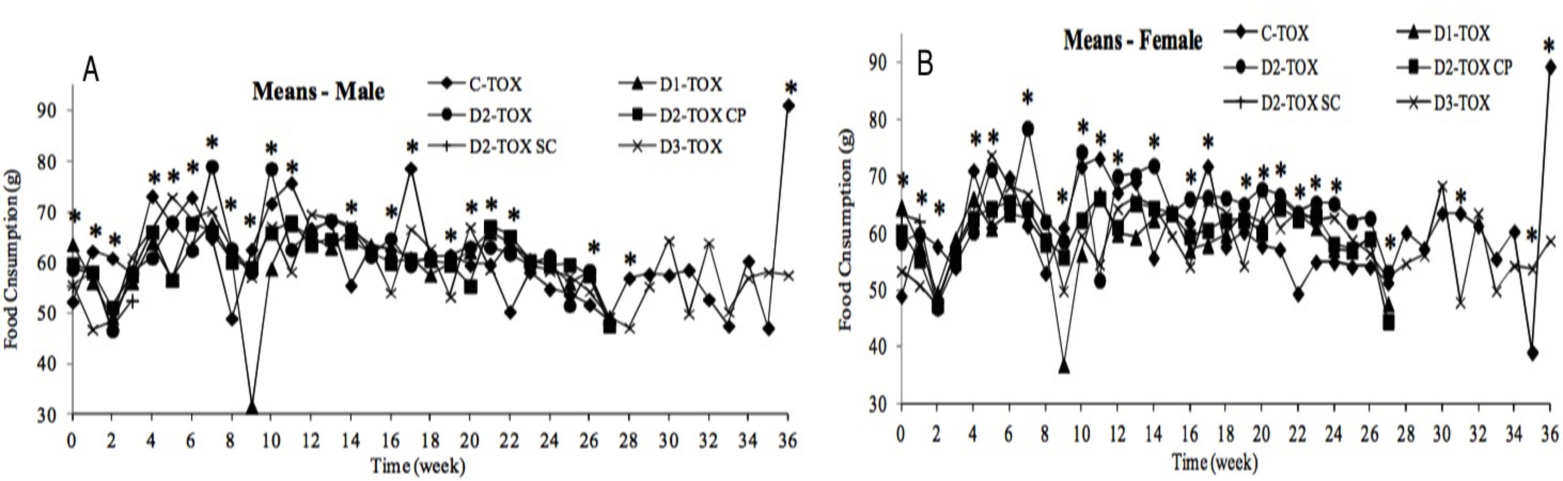
Food consumption–TOX groups. (A)–males. (B)–females.

The hamsters’ food consumption was recorded weekly during the acclimation and administration periods (Fig 3). The average food consumption in the course of the acclimation was expressed as baseline. The mean food consumption slightly decreased after the first administration in most groups. Thereafter, a high variability in food consumption was observed in all groups. Although there were many statistically significant differences between groups in many study weeks, due to high variability no clear trends indicating treatment related changes in food consumption were found. No influence of ONCOS-102 on the hamsters’ food consumption in the course of the study administration period was observed.

#### Hematology and clinical chemistry

Blood samples for hematology and clinical chemistry examinations were collected from all the animals on day 7 (Examination 1, before the first ONCOS-102 administration), on day 29 (Examination 2), on day 190 (Examination 3, end of the administration period) and on day 255 (S4 and S5 Tables).

Hematology: there were no changes in hematology parameters after the ONCOS-102 administration, except for the significantly higher values of MCV, MCH and MCHC in group D2-TOX CP as compared to control in Examination 3, most probably caused by additive item (Cyclophosphamide).

Clinical chemistry: no significant changes were observed in the mean serum activity of the liver enzymes such as Lactate dehydrogenase (LDH), Alanine aminotransferase (ALT) and Aspartate aminotransferase (AST), except for the statistically significant higher activity of LDH in the D3-TOX recovery males as compared to control recovery animals (Examination 4, p<0.001). A slight decrease (up to 10% and within reference limits) in the mean total protein (TP) values was recorded in dosed females (with statistically significance in the D1-TOX (p<0.05), D2-TOX CP (p<0.05) and D3-TOX females (p<0.01)) on day 190 (Examination 3) as compared to control. This finding corresponded with the statistically significant decrease in the mean globulin values (Glo) in the D1-TOX (p<0.05) and D2-TOX CP (p<0.01) females. No statistically significant differences were found in dosed males on Day 190.

No considerable changes were observed in the mean liver enzymes serum activity during the study. No dose-related changes of the serum proteins were observed in dosed males. Although the decrease in the serum proteins was recorded in dosed females on Day 190, all the mean values varied within the reference limits and could be evaluated as biologically insignificant.

#### Neutralizing antibodies in hamster’s sera

All virus-treated animals developed NAbs against the virus (S1–S3 Figs). Dosing had an effect on the Nab formation, since the NAb blocking effect in animals from group D1-TOX was statistically smaller (p<0.05 –p<0.001) in comparison to the groups receiving 10-fold or 100-fold larger dose of virus (D2-TOX and D3-TOX), respectively (S2 Fig). The latter groups had no statistically significant difference when compared against each other. This possibly indicates some level of NAb saturation, where the amount of NAbs increases in relation to the dose used up to a certain point, but after reaching a certain limit the level of NAbs does no longer rise following the same kinetics even if more virus is dosed. Overall, as expected, when compared to the other virus-injected groups, D3-TOX had the highest levels of NAbs, even though the statistically significant difference (p<0.01 –p<0.001) was only observed against D1-TOX receiving 100-fold less virus, but not the group receiving 10-fold less virus (D2-TOX), (S2 Fig). NAb levels at different time points were further studied with the highest virus treatment dose (D3-TOX) by including a late time point of 255 days (S1 Fig). These results indicate that NAbs already reached high expression levels and activity 29 days after virus treatment. However, increased levels of NAbs were formed by day 190, and the levels remained high until end of the experiment at Day 255. Both adding cyclophosphamide or changing the injection route to subcutaneous decreased NAb formation when compared to animals treated with same adenoviral dose without CP and with default virus injection route (D2-TOX), (S3 Fig). This indicates that combination therapy with CP or changing the administration route of adenovirus may alter NAb formation.

### Pathology

Gross pathology findings are presented in S6 and S7 Tables.

Liver: focal areas of mononuclear cell infiltration in the liver parenchyma were noticed both in some control and the treated animals, therefore without relation to the treatment. A surprising feature was irregular distribution of this lesion in different liver lobes. The cause of this lesion is unclear, but due to the presence of this lesion in five control animals its relation to the ONCOS-102 could be excluded.

Kidneys: the findings in the kidneys concerned both control and administered hamsters (cortical scars, focal tubular dilatation, focal tubular basophilia, medullar mineralization, hyaline casts) were without relation to the treatment.

Lungs: histopathological examination found encapsulated hematoma with marginal resorption and frequently with focal calcification. This lesion was in relation to the intracardial injection and subsequent hemorrhage into the lungs or thoracic cavity. Similarly, the pleural adhesions and pleural fibrosis found in a few animals are a consequence of traumatic injury caused by previous intracardial injection.

Male and female genital tract: the testes of three D1-TOX, three D2-TOX, four D2-TOX CP, and one D3-TOX males showed adhesions of different extent to parietal layer of tunica vaginalis accompanied by red- brown or yellowish color in two D2-TOX CP males. Reduced size of testes was recorded in seven administered males. Similar adhesions and yellowish color were observed in epididymides of two males from group D2-TOX SC and one male of group D1-TOX. In one D3-TOX male, the epididymidis was enlarged, and in one D1-TOX this organ was reduced in size.

All findings in the female genital tract were in relation to the advanced age of females, without relation to the ONCOS-102.

Miscellaneous findings: all findings in the heart, except for spontaneous lipomatous atrophy in one case, together with the findings in the mediastinum, retroperitoneum, abdominal cavity and mesentery were a consequence of previous intracardial or intraperitoneal injection. Their relation to the substance tested is secondary.

ONCOS-102 in the doses used did not cause gross or histopathological changes in the liver and kidneys of surviving animals indicative of a toxic effect.

Other lesions found in the treated animals were either of spontaneous character or they were not in direct relation to the ONCOS-102 administration.

### Bio-distribution study

**Fig 4:**
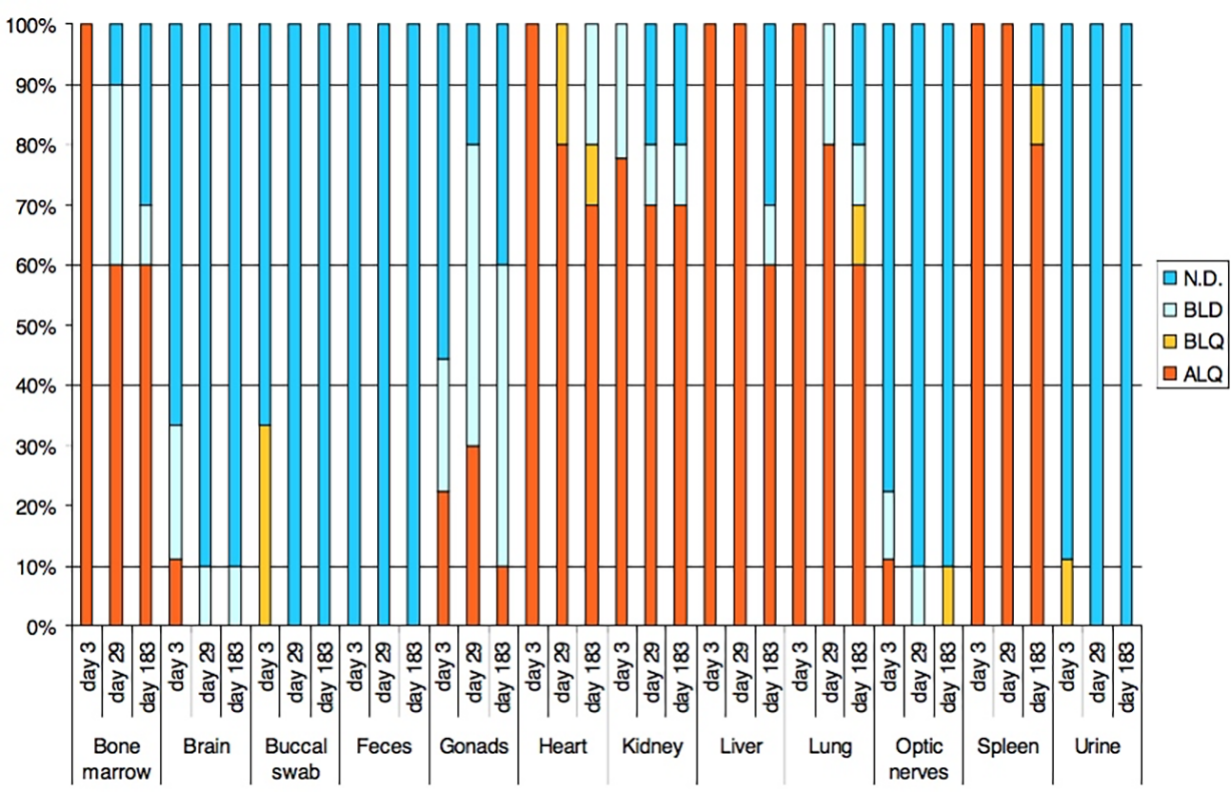
Portions of final results in days and treatment groups. ALQ–samples with detection of adenoviral sequence above limit of quantification (positive samples), BLQ–below limit of quantification, BLD–below limit of detection, N.D.–not detected.

**Fig 5:**
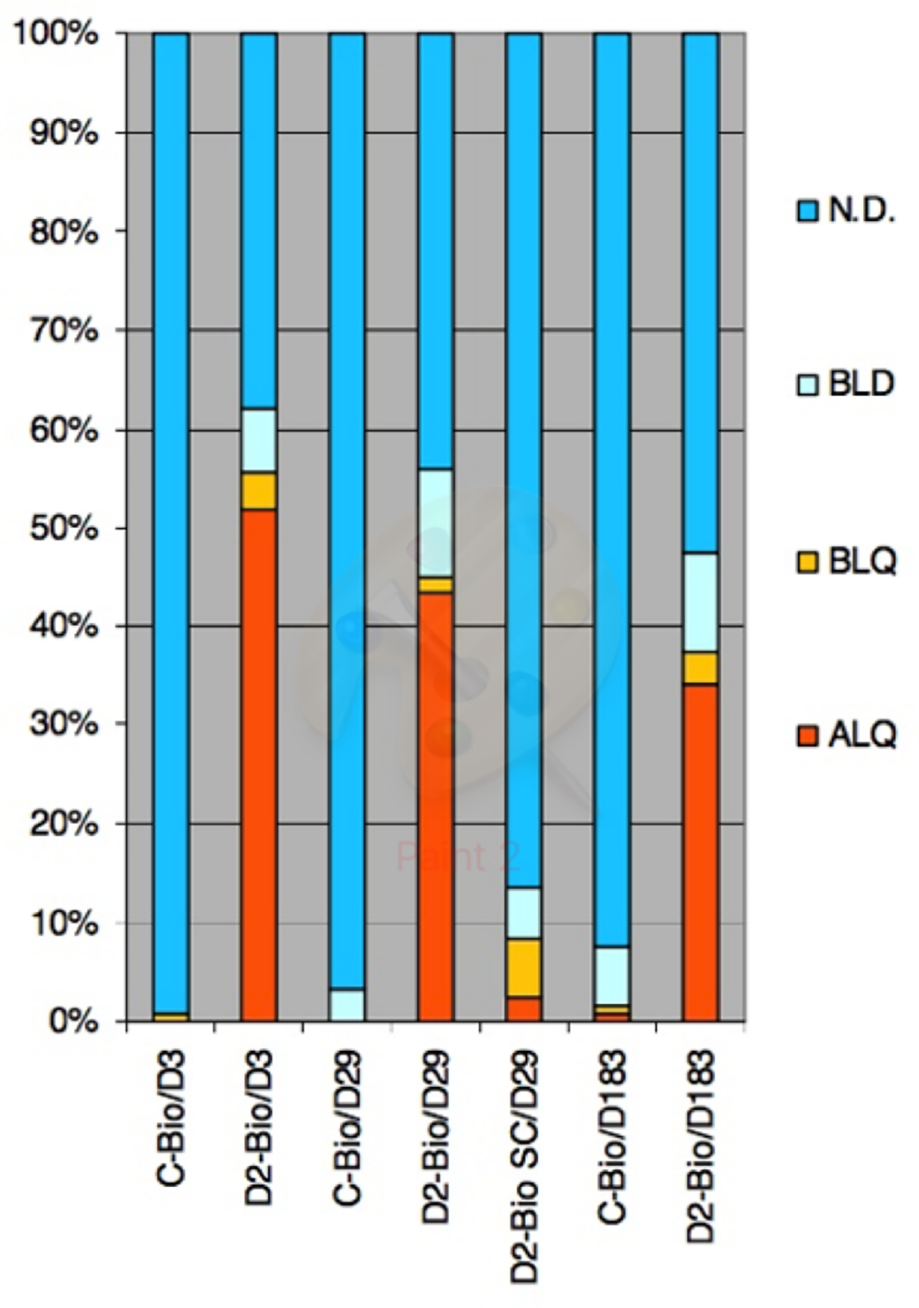
Portions of final results in sample types from D2-Bio group in days 3, 29 and 183. ALQ–samples with detection of adenoviral sequence above limit of quantification (positive samples), BLQ–below limit of quantification, BLD–below limit of detection, N.D.–not detected.

There was observed overall decrease of concentration of viral DNA from day 3 to Day 29 and to Day 183. The total portion of quantifiable samples together with samples below LOQ was 55,6%, 45,0% and 37,5% in day 3, day 29 and day 183 of D2-Bio group respectively (Figs 4 and 5). The concentration of viral DNA is plotted in S4 Fig.

**Fig 6:**
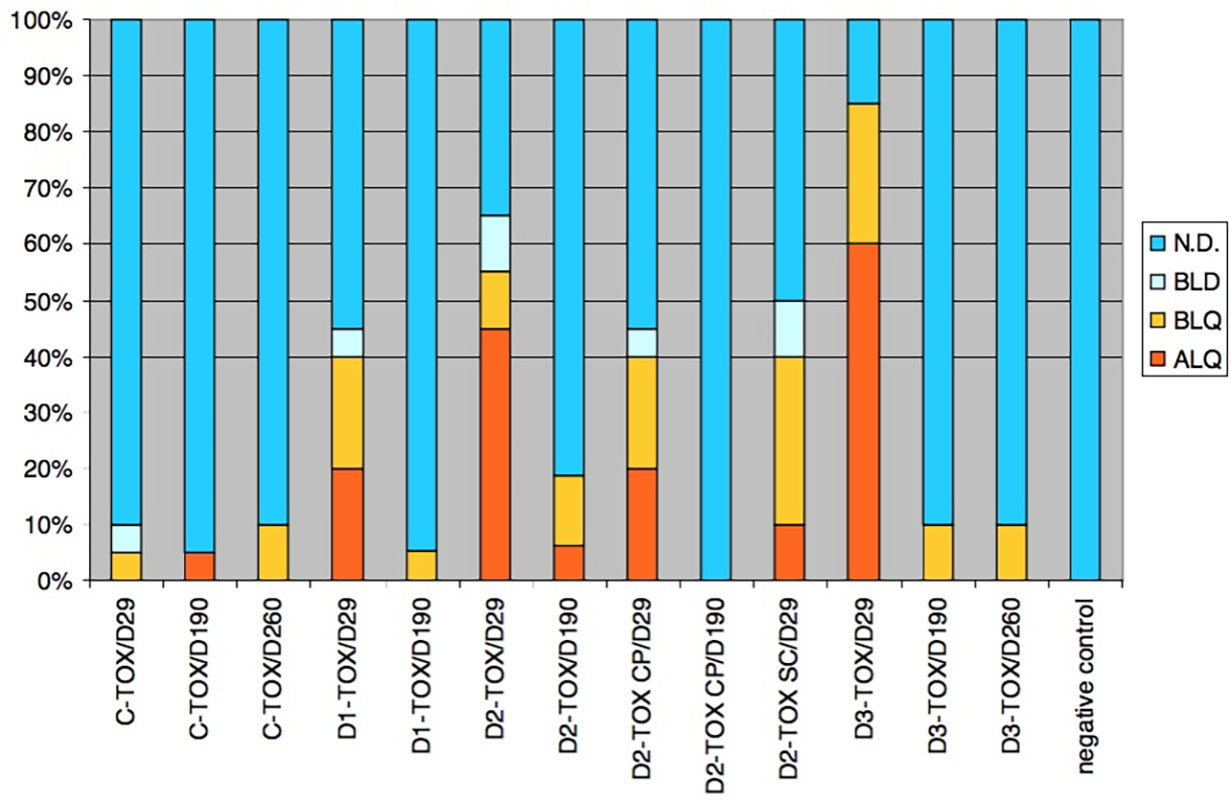
Portions of final results in samples of serum from TOX groups in day 29, 190 and 260. ALQ–samples with detection of adenoviral sequence above limit of quantification (positive samples), BLQ–below limit of quantification, BLD–below limit of detection, N.D.–not detected.

The highest accumulation of viral particles was observed in heart, liver, lung, spleen, kidney and bone marrow. The Kruskal-Wallis test and Dunn's Multiple Comparison Test showed significant changes (p<0.05) of viral DNA concentration in heart, liver, lung and spleen. Excepting spleen the significant changes were registered between day 3 and day 29 and between day 3 and 183. The significant change in spleen was registered only between Day 3 and Day183. In bone marrow and kidney was not registered significant change. The concentration in these tissues was lower than 10 copies per ng of DNA. The distribution of virus in animals from group D2-Bio SC is very limited in comparison to the group D2-Bio. The content of viral DNA in serum samples from day 29 was low. The number of positive samples and samples bellow LOQ was 40%, 55% and 85% in D1-TOX, D2-TOX and D3-TOX group respectively (Fig 6). The number of positive samples and samples bellow LOQ in D2-TOX CP and D2-TOX SC was 40%.

## Discussion

Metastatic tumors exhibit resistance to standard therapies, therefore new and more effective treatment modalities are in high need [1, 2].

Oncolytic adenoviruses have shown a potential for the treatment of cancer. They have been tested extensively in hundreds of preclinical studies and clinical trials. Importantly the safety of adenoviral cancer gene therapy has been very well studied and considered a safe therapeutic system [10–12], but only 2 products [13, 14] have been approved thus far—mainly due to low anti-cancer efficacy. Therefore new approaches are focused on improving the efficacy by combinatory treatment options with chemotherapy [15–17], immune checkpoint inhibitors [18, 19], and radiotherapy [13, 20].

The aim of this study was to evaluate the safety profile of the ONCOS-102 after administration in Syrian hamsters. Study was performed to support clinical trial application of ONCOS-102 (NCT01598129) for therapy of advanced cancers.

ONCOS-102 is an oncolytic adenovirus, designed to act as anti-cancer agent, by exhibiting dual mode of action: i) to selectively kill tumor cells and ii) to induce anti-tumor immune responses. Its anticancer efficacy has been already shown in numerous pre-clinical studies on melanoma, soft tissue sarcoma and safety has been assessed in Phase I study (NCT01598129) [8, 21]. Administration of ONCOS-102 for advanced solid tumor patients resulted in prominent infiltration of CD8+ lymphocytes, and induction of Th1-type polarization in the tumor microenvironment [8, 21].

Human adenovirus serotype 5 is a species-specific virus. It does not replicate to the same extent as in humans in most animal models. However, Syrian hamsters have been reported as semi-permissive to human adenovirus [22]. Therefore, it is a suitable animal model for toxicological and bio-distribution studies with replicating adenovirus. This is also the position taken by the national authorities in the USA and Europe, thus hamster was selected as a relevant animal model for toxicity testing of ONCOS-102 in this study. The injection routes were selected for the following reasons: systemic administration is considered the most toxic, i.e. this route of administration, if any, would be the most likely to lead to toxicity. However, in hamsters, reliable i.v. injection is difficult and thus i.c. was used as the best-available form of systemic delivery. However, this cannot be performed repeatedly and thus the treatment was continued with i.p. injections. Since the animals had no tumours, i.t. administration was not an option and i.p. injection was considered the next best choice to simulate the overall toxicity and biodistribution profile achievable with i.t. injection.

The treatment with ONCOS-102 did not cause any adverse effects on body weight and food consumption. Additionally, studies showed no considerable differences in hematology and clinical chemistry parameters in treated groups when compared to control animals after 26 weeks of the study.

Presented overall findings do not surprise, since extensive clinical and non-clinical evidence demonstrate benign safety profile of adenovirus. Hundreds of cancer patients have been treated with replicating competent serotype 5 adenovirus Onyx-015 (E1B attenuated) in numerous clinical trials [23, 24]. Onyx-015 was generally well tolerated at doses of up to 2×10^12^ particles by intratumoral, intraperitoneal, hepatic arterial and intravenous administration. No dose limiting toxicities (DLTs) have been identified by any route of administration. Flu-like symptoms (fever, rigors) were the most typical toxicities and were mostly seen in cancer patients treated intravascularly [24]. Similar observations with no DLTs have been reported in a Phase 1 study of 18 melanoma patients receiving combination therapy with T-VEC and anti-CTLA-4 antibody [25]. No dose-limiting toxicity or maximum tolerated dose (MTD) has been noticed as well in Phase 1 clinical trial of intratumoral infusion of reovirus for the treatment of recurrent malignant gliomas [26].

Indeed thousands of patients were treated in many trials testing HSV, adenovirus, Reolysin and very importantly all studies were without any major virus-related complication, and the assessment of DLT and MTD has been noticed in any trial [27].

We conclude that under the test conditions used, the repeated administration of hamsters with ONCOS-102 was well tolerated, without significant signs indicating toxic effects.

## Supporting information

S1 Table Hematology parameters. (DOC)

S2 Table Clinical chemistry parameters. * Day 3 and 190 ** Day 255.
(DOCX)

S3 Table Histopathology organ and tissue list. (DOCX)

S4 Table Gross pathology findings (day 29) summary. -                organ examined, no pathological findings
/                organ not examined
NA                not applicable
GRADE 1        minimal/very few/very small
GRADE 2        slight/few/small
GRADE 3        moderate/moderate number/moderate size
GRADE 4        marked/many/large
)                finding unilateral in paired organs
P                finding present, severity not scored
(DOCX)

S5 Table Gross pathology findings (day 190 and day 256) summary. -                organ examined, no pathological findings
/                organ not examined
NA                not applicable
GRADE 1        minimal/very few/very small
GRADE 2        slight/few/small
GRADE 3        moderate/moderate number/moderate size
GRADE 4        marked/many/large
)                finding unilateral in paired organs
P                finding present, severity not scored
(DOCX)

S6 Table Hematology analysis. Statistically significant difference at the 95.0% confidence level is pointed up in boldx Normality test not passed, which tends to invalidate the tests comparing the standard deviations* Statistically significant difference only between means test groups D1-TOX, D2-TOX, D2-TOX CP, D2-TOX SC or D3-TOX versus control group C-TOX** Statistically significant difference only between medians test groups D1-TOX, D2-TOX, D2-TOX CP, D2-TOX SC or D3-TOX versus control group C-TOX*** Statistically significant difference between means and medians test groups D1-TOX, D2-TOX, D2-TOX CP, D2-TOX SC or D3-TOX versus control group C-TOX+ statistically significant difference among standard deviations (F test)# no variablity in one of the columns
(DOCX)

S7 Table Clinical chemistry analysis. Statistically significant difference at the 95.0% confidence level is pointed up in boldx Normality test not passed, which tends to invalidate the tests comparing the standard deviations* Statistically significant difference only between means test groups D1-TOX, D2-TOX, D2-TOX CP, D2-TOX SC or D3-TOX versus control group C-TOX** Statistically significant difference only between medians test groups D1-TOX, D2-TOX, D2-TOX CP, D2- TOX SC or D3-TOX versus control group C-TOX*** Statistically significant difference between means and medians test groups D1-TOX, D2-TOX, D2-TOX CP, D2-TOX SC or D3-TOX versus control group C-TOX+ statistically significant difference among standard deviations (F test)# no variablity in one of the columns
(DOCX)

S1 Fig NAb activity in serum at 29 and 190 days after starting the treatments. The lowest, medium and highest dose of virus treatment escalated from 1 to 100-fold (D1-TOX 1x; D2-TOX 10x and D3-TOX 100x). Bars present NAb blocking effect ± SEM. Statistical analyses was performed with LSD post hoc comparisons test. Horizontal lines indicate statistically significant differences between sampling days within a group. Stars indicate statistical significance between treatment groups D1-TOX vs. D2-TOX/D3-TOX (*** = P≤ 0.001; ** = P≤ 0.01 and * = P≤ 0.05). There was no statistically significant difference between D2-TOX and D3-TOX groups either sampling days.
(TIFF)

S2 Fig NAb activity in hamsters’ serum at 29, 190 and 255 days after the highest dose of virus treatment (D3-TOX 100x). Bars present NAb blocking effect ± SEM. Statistical analyses has been performed with LSD post hoc comparisons test.
(TIFF)

S3 Fig NAb activity in hamsters serum after 29 days treatment with medium (10x) dose of adenovirus either intracardially/intraperitoneally (D2-TOX), with cyclophosphamide added (D2-TOX CP) or subcutaneously (D2-TOX SC). Bars present NAb blocking effect ± SEM. Statistical analyses has been performed with LSD post hoc comparisons test (*** = P≤ 0.001; ** = P≤ 0.01 and * = P≤ 0.05).
(TIFF)

S4 Fig Medians of logarithms of ratio Ad5/ng DNA in tissues from Day 3, 29 and 183 of D2-Bio group. Brain, Gonads, Optic nerves, Feces, Buccal swabs and Urine are not plotted. The whiskers represent standard deviation.
(TIF)

## References

[1] Diaconu I, Cerullo V, Hirvinen ML, Escutenaire S, Ugolini M, Pesonen SK, et al Immune response is an important aspect of the antitumor effect produced by a CD40L-encoding oncolytic adenovirus. Cancer research. 2012;72(9):2327–38. doi: 10.1158/0008-5472.CAN-11-2975 . 22396493 10.1158/0008-5472.CAN-11-2975

[2] Gilboa E. How tumors escape immune destruction and what we can do about it. Cancer Immunology, Immunotherapy. 1999;48(7):382–5. doi: 10.1007/s002620050590 10501851 10.1007/s002620050590 PMC11037178

[3] Koski A, Kangasniemi L, Escutenaire S, Pesonen S, Cerullo V, Diaconu I, et al Treatment of cancer patients with a serotype 5/3 chimeric oncolytic adenovirus expressing GMCSF. Molecular therapy: the journal of the American Society of Gene Therapy. 2010;18(10):1874–84. doi: 10.1038/mt.2010.161 ; PubMed Central PMCID: PMC2951567. 20664527 10.1038/mt.2010.161 PMC2951567

[4] Pesonen S, Kangasniemi L, Hemminki A. Oncolytic adenoviruses for the treatment of human cancer: focus on translational and clinical data. Molecular pharmaceutics. 2011;8(1):12–28. doi: 10.1021/mp100219n . 21126047 10.1021/mp100219n

[5] Pesonen S, Nokisalmi P, Escutenaire S, Sarkioja M, Raki M, Cerullo V, et al Prolonged systemic circulation of chimeric oncolytic adenovirus Ad5/3-Cox2L-D24 in patients with metastatic and refractory solid tumors. Gene therapy. 2010;17(7):892–904. doi: 10.1038/gt.2010.17 . 20237509 10.1038/gt.2010.17

[6] Dranoff G. GM-CSF-based cancer vaccines. Immunological Reviews. 2002;188(1):147–54. doi: 10.1034/j.1600-065X.2002.18813.x 12445288 10.1034/j.1600-065x.2002.18813.x

[7] van de Laar L, Coffer PJ, Woltman AM. Regulation of dendritic cell development by GM-CSF: molecular control and implications for immune homeostasis and therapy. Blood. 2012;119(15):3383–93. doi: 10.1182/blood-2011-11-370130 . 22323450 10.1182/blood-2011-11-370130

[8] Ranki T, Joensuu T, Jäger E, Karbach J, Wahle C, Kairemo K, et al Local treatment of a pleural mesothelioma tumor with ONCOS-102 induces a systemic antitumor CD8+T-cell response, prominent infiltration of CD8+lymphocytes and Th1 type polarization. OncoImmunology. 2014;3(10):e958937 doi: 10.4161/21624011.2014.958937 25941579 10.4161/21624011.2014.958937 PMC4292415

[9] Sarkioja M, Pesonen S, Raki M, Hakkarainen T, Salo J, Ahonen MT, et al Changing the adenovirus fiber for retaining gene delivery efficacy in the presence of neutralizing antibodies. Gene therapy. 2008;15(12):921–9. doi: 10.1038/gt.2008.56 . 18401431 10.1038/gt.2008.56

[10] Koski A, Rajecki M, Guse K, Kanerva A, Ristimaki A, Pesonen S, et al Systemic adenoviral gene delivery to orthotopic murine breast tumors with ablation of coagulation factors, thrombocytes and Kupffer cells. J Gene Med. 2009;11(11):966–77. doi: 10.1002/jgm.1373 . 19670332 10.1002/jgm.1373

[11] Freytag SO, Stricker H, Movsas B, Kim JH. Prostate cancer gene therapy clinical trials. Molecular therapy: the journal of the American Society of Gene Therapy. 2007;15(6):1042–52. doi: 10.1038/sj.mt.6300162 . 17406342 10.1038/sj.mt.6300162

[12] Lubaroff DM, Konety BR, Link B, Gerstbrein J, Madsen T, Shannon M, et al Phase I clinical trial of an adenovirus/prostate-specific antigen vaccine for prostate cancer: safety and immunologic results. Clin Cancer Res. 2009;15(23):7375–80. doi: 10.1158/1078-0432.CCR-09-1910 ; PubMed Central PMCID: PMCPMC2787649. 19920098 10.1158/1078-0432.CCR-09-1910 PMC2787649

[13] 10.1016/j.athoracsur.2005.01.048.

[14] Yamamoto M, Curiel DT. Current issues and future directions of oncolytic adenoviruses. Molecular therapy: the journal of the American Society of Gene Therapy. 2010;18(2):243–50. doi: 10.1038/mt.2009.266 ; PubMed Central PMCID: PMCPMC2839292. 19935777 10.1038/mt.2009.266 PMC2839292

[15] Nguyen A, Ho L, Wan Y. Chemotherapy and Oncolytic Virotherapy: Advanced Tactics in the War against Cancer. Front Oncol. 2014;4:145 doi: 10.3389/fonc.2014.00145 ; PubMed Central PMCID: PMCPMC4052116. 24967214 10.3389/fonc.2014.00145 PMC4052116

[16] Siurala M, Bramante S, Vassilev L, Hirvinen M, Parviainen S, Tahtinen S, et al Oncolytic adenovirus and doxorubicin-based chemotherapy results in synergistic antitumor activity against soft-tissue sarcoma. International journal of cancer Journal international du cancer. 2015;136(4):945–54. doi: 10.1002/ijc.29048 . 24975392 10.1002/ijc.29048

[17] Liikanen I, Ahtiainen L, Hirvinen ML, Bramante S, Cerullo V, Nokisalmi P, et al Oncolytic adenovirus with temozolomide induces autophagy and antitumor immune responses in cancer patients. Molecular therapy: the journal of the American Society of Gene Therapy. 2013;21(6):1212–23. doi: 10.1038/mt.2013.51 ; PubMed Central PMCID: PMCPMC3681222. 23546299 10.1038/mt.2013.51 PMC3681222

[18] Woller N, Gürlevik E, Fleischmann-Mundt B, Knocke S, Geffers R, Manns MP, et al Virotherapy overcomes tumor resistance to PD-1-immunotherapy by broad mutanome-directed T cell responses in mice. Zeitschrift für Gastroenterologie. 2015;53(01). doi: 10.1055/s-0034-1397203 10.1038/mt.2015.115 PMC4817928 26112079

[19] Vile RG. How to train your oncolytic virus: the immunological sequel. Molecular therapy: the journal of the American Society of Gene Therapy. 2014;22(11):1881–4. doi: 10.1038/mt.2014.188 ; PubMed Central PMCID: PMCPMC4429744. 25365984 10.1038/mt.2014.188 PMC4429744

[20] Touchefeu Y, Vassaux G, Harrington KJ. Oncolytic viruses in radiation oncology. Radiother Oncol. 2011;99(3):262–70. doi: 10.1016/j.radonc.2011.05.078 . 21704402 10.1016/j.radonc.2011.05.078

[21] Vassilev L, Ranki T, Joensuu T, Jager E, Karbach J, Wahle C, et al Repeated intratumoral administration of ONCOS-102 leads to systemic antitumor CD8 T-cell response and robust cellular and transcriptional immune activation at tumor site in a patient with ovarian cancer. Oncoimmunology. 2015;4(7):e1017702 doi: 10.1080/2162402X.2015.1017702 ; PubMed Central PMCID: PMC4485730. 26140248 10.1080/2162402X.2015.1017702 PMC4485730

[22] Thomas MA, Spencer JF, La Regina MC, Dhar D, Tollefson AE, Toth K, et al Syrian hamster as a permissive immunocompetent animal model for the study of oncolytic adenovirus vectors. Cancer research. 2006;66(3):1270–6. doi: 10.1158/0008-5472.CAN-05-3497 . 16452178 10.1158/0008-5472.CAN-05-3497

[23] Nemunaitis J, Cunningham C, Buchanan A, Blackburn A, Edelman G, Maples P, et al Intravenous infusion of a replication-selective adenovirus (ONYX-015) in cancer patients: safety, feasibility and biological activity. Gene therapy. 2001;8(10):746–59. http://dx.doi.org/10.1038/sj.gt.3301424. doi: 10.1038/sj.gt.3301424 . 11420638 10.1038/sj.gt.3301424

[24] Kirn D. Clinical research results with dl1520 (Onyx-015), a replication-selective adenovirus for the treatment of cancer: what have we learned? Gene therapy. 2001;8:89–98. doi: 10.1038/sj.gt.3301377 11313778 10.1038/sj.gt.3301377

[25] Turnbull S, West EJ, Scott KJ, Appleton E, Melcher A, Ralph C. Evidence for Oncolytic Virotherapy: Where Have We Got to and Where Are We Going? Viruses. 2015;7(12):6291–312. doi: 10.3390/v7122938 ; PubMed Central PMCID: PMCPMC4690862. 26633468 10.3390/v7122938 PMC4690862

[26] Kicielinski KP, Chiocca EA, Yu JS, Gill GM, Coffey M, Markert JM. Phase 1 clinical trial of intratumoral reovirus infusion for the treatment of recurrent malignant gliomas in adults. Molecular therapy: the journal of the American Society of Gene Therapy. 2014;22(5):1056–62. http://www.nature.com/doifinder/10.1038/mt.2014.21. doi: 10.1038/mt.2014.21 ; PubMed Central PMCID: PMCPMC4015229. 24553100 10.1038/mt.2014.21 PMC4015229

[27] Wollmann G, Ozduman K, van den Pol AN. Oncolytic virus therapy for glioblastoma multiforme: concepts and candidates. Cancer J. 2012;18(1):69–81. doi: 10.1097/PPO.0b013e31824671c9 ; PubMed Central PMCID: PMCPMC3632333. 22290260 10.1097/PPO.0b013e31824671c9 PMC3632333

