# Supplementary material for: Toxicological and bio-distribution profile of a GM-CSF-expressing, double-targeted, chimeric oncolytic adenovirus ONCOS-102 – Support for clinical studies on advanced cancer treatment
Source: PLoS One. 2017 Aug 10;12(8):e0182715. doi: 10.1371/journal.pone.0182715 (PMC5552138; doi:10.1371/journal.pone.0182715)
Supplement: S1 Table — (DOC) [file pone.0182715.s001.doc]

| **Parameter** | **Name of instrument/Method** |   **Unit of measure (SI)** |
| --- | --- | --- |
| **Erythrocyte count** | Vet ABC Animal Blood Counter, ABX Diagnostics | 10^12^/l |
| **Haemoglobin** | Vet ABC Animal Blood Counter, ABX Diagnostics | g/l |
| **Haematocrit** | Vet ABC Animal Blood Counter, ABX Diagnostics | l/l |
| **Mean corpuscular volume** | Vet ABC Animal Blood Counter, ABX Diagnostics | fl |
| **Mean corpuscular haemoglobin** | Vet ABC Animal Blood Counter, ABX Diagnostics | pg |
| **Mean corpuscular haemoglobin concentration** | Vet ABC Animal Blood Counter, ABX Diagnostics | g/l |
| **Leukocyte count** | Vet ABC Animal Blood Counter, ABX Diagnostics | 10^9^/l |
| **Platelet count** | Vet ABC Animal Blood Counter, ABX Diagnostics | 10^9^/l |
| **Nucleated RBC** | Microscopic, peripheral blood smears stained by May-Grunwald and Giemsa-Romanovski | ‰ |
| **Differential leukocyte count** | Microscopic, peripheral blood smears stained by May-Grunwald and Giemsa-Romanovski | 10^9^/l |
| **Activated partial thromboplastin time** | Coagulometer STart 4 (Diagnostica Stago), PTT automate | sec. |
| **Prothrombin time** | Coagulometer STart 4 (Diagnostica Stago), NEOPLASTINE® CL PLUS | sec. |
