# Supplementary material for: Toxicological and bio-distribution profile of a GM-CSF-expressing, double-targeted, chimeric oncolytic adenovirus ONCOS-102 – Support for clinical studies on advanced cancer treatment
Source: PLoS One. 2017 Aug 10;12(8):e0182715. doi: 10.1371/journal.pone.0182715 (PMC5552138; doi:10.1371/journal.pone.0182715)
Supplement: S2 Table — * Day 3 and 190 ** Day 255. (DOCX) [file pone.0182715.s002.docx]

| **Parameter** | **Name of instrument/Method** | **Unit of measure (SI)** |
| --- | --- | --- |
| Alanine amino transferase | Dimension RxL Max Dade Behring, Behring Dimension ALT* Dimension Vista® 500, Siemens Healthcare Diagnostics/Dimension ALT** | μkat/l |
| Aspartate amino transferase | Dimension RxL Max Dade Behring, Behring Dimension AST* Dimension Vista® 500, Siemens Healthcare Diagnostics/Dimension AST** | μkat/l |
| Lactate | Dimension RxL Max Dade Behring, Behring Dimension LD* | μkat/l |
| Dehydrogenase | Dimension Vista® 500, Siemens Healthcare Diagnostics/Dimension LD** | 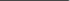 |
| Protein total | Dimension RxL Max Dade Behring , Behring Dimension TP* Dimension Vista® 500, Siemens Healthcare Diagnostics/Dimension TP** | g/l   |
| Albumin | Dimension RxL Max Dade Behring , Behring Dimension ALB* Dimension Vista® 500, Siemens Healthcare Diagnostics/Dimension ALB** | g/l |
| Globulin | Calculated |   g/l |
| Albumin/Globulin ratio | Calculated | - |
