# Supplementary material for: Toxicological and bio-distribution profile of a GM-CSF-expressing, double-targeted, chimeric oncolytic adenovirus ONCOS-102 – Support for clinical studies on advanced cancer treatment
Source: PLoS One. 2017 Aug 10;12(8):e0182715. doi: 10.1371/journal.pone.0182715 (PMC5552138; doi:10.1371/journal.pone.0182715)
Supplement: S3 Table — (DOCX) [file pone.0182715.s003.docx]

| **Tissue** |   **Fix** |   **Slide Preparation** |   **Microscopic Examination** |
| --- | --- | --- | --- |
| Adrenal glands | x |  |  |
| Aorta | x |  |  |
| Bone & bone marrow (femur) | x |  |  |
| Brain | x | x | x |
| Cecum | x |  |  |
| Esophagus | x |  |  |
| Epididymides | x | x | x |
| Eyes (incl. optic nerves) | x |  |  |
| Esophagus | x |  |  |
| Gall bladder | x |  |  |
| Harderian gland | x |  |  |
| Heart | x | x | x |
| Large & small intestine | x |  |  |
| Kidneys | x | x | x |
| Liver | x | x | x |
| Lungs (incl. mainstem bronchi) | x | x | x |
| Mammary gland | x |  |  |
| Mesenteric (and any abnormal) lymph nodes | x |  |  |
| Ovaries | x | x | x |
| Pancreas | x |  |  |
| Penis | x |  |  |
| Peripheral nerve | x |  |  |
| Pituitary | x |  |  |
| Prostate | x | x | x |
| Salivary gland | x |  |  |
| Seminal vesicles | x |  |  |
| Skeletal muscle | x |  |  |
| Skin | x |  |  |
| Spinal cord | x |  |  |
| Spleen | x | x | x |
| Sternum | x |  |  |
| Stomach | x |  |  |
| Testes | x | x | x |
| Thymus | x |  |  |
| Thyroids (incl. parathyroid) | x |  |  |
| Trachea | x |  |  |
| Urinary bladder | x |  |  |
| Uterus | x | x | x |
| Vagina | x |  |  |
| All gross lesions | x |  |  |
