# Supplementary material for: Toxicological and bio-distribution profile of a GM-CSF-expressing, double-targeted, chimeric oncolytic adenovirus ONCOS-102 – Support for clinical studies on advanced cancer treatment
Source: PLoS One. 2017 Aug 10;12(8):e0182715. doi: 10.1371/journal.pone.0182715 (PMC5552138; doi:10.1371/journal.pone.0182715)
Supplement: S4 Table — - organ examined, no pathological findings / organ not examined NA not applicable GRADE 1 minimal/very few/very small GRADE 2 slight/few/small GRADE 3 moderate/moderate number/moderate size GRADE 4 marked/many/large ) finding unilateral in paired organs P finding present, severity not scored (DOCX) [file pone.0182715.s004.docx]

| **Dose group** | **C-TOX** | | **D1-TOX** | | **D2-TOX** | | **D2-TOX CP** | | **D2-TOX SC** | | **D3-TOX** | |
| --- | --- | --- | --- | --- | --- | --- | --- | --- | --- | --- | --- | --- |
| **Sex** | **M** | **F** | **M** | **F** | **M** | **F** | **M** | **F** | **M** | **F** | **M** | **F** |
| **Number of animals** | **10** | **10** | **10** | **10** | **10** | **10** | **10** | **10** | **5** | **5** | **10** | **10** |
| **Organ findings** |  |  |  |  |  |  |  |  |  |  |  |  |
| **Autolysis Epididymides**  -adhesion  -yellowish colour  **Heart**  -brown content in pericardial cavity  -brown-green nodule on pericardium  -hemopericardium  **Kidneys**  -pale colour  -red focus  **Liver**  -marked structure  -pale colour  **Lungs**  -brown-green nodule  -focal hemorrhage  -venostasis  **Lymph node cervical**  -enlarged  -hemorrhage  **Mediastinum**  -brown-green nodule  **Ovary**  -cyst  **Spleen**  -enlarged  **Stomach**  -black-brown foci  **Testes**  -adhesion  -flaccid  -red-brown colour  -yellowish colour  **Thoracic cavity**  -brown-green nodule  -hemothroax  **Thymus**  -atrophy  **Urinary bladder**  -dilatiation  **Uterus**  - dilatiation | -  -  -  -  -  -  -  -  -  -  -  2  -  1  -  1  NA  -  -  -  -  -  -  -  -  8  -  NA | -  NA  NA  -  -  1  -  -  -  1  -  1  -  -  1  -  -  -  1  NA  NA  NA  NA  -  1  9  -  7 | -  -  -  -  2  -  -  -  -  -  -  1  -  -  -  -  NA  -  -  2  -  -  -  1  -  10  -  NA | -  NA  NA  -  -  -  -  -  -  -  -  1  -  -  -  -  -  -  -  NA  NA  NA  NA  -  -  10  -  5 | -  -  -  -  -  -  -  1  -  -  1  -  -  -  -  -  NA  -  -  2  1  -  -  1  -  10  -  NA | 1  NA  NA  -  -  -  1  -  -  -  -  3  1  -  -  1  -  -  1  NA  NA  NA  NA  1  -  10  1  1 | -  -  -  -  -  -  1  -  1  -  -  5  -  -  -  -  NA  1  -  3  1  1  1  -  -  10  -  NA | -  NA  NA  -  -  -  1  -  1  1  -  -  -  -  -  -  -  -  -  NA  NA  NA  NA  -  -  10  -  - | -  2  2  -  -  -  -  -  -  -  -  -  -  -  -  -  NA  -  -  -  -  -  -  -  -  5  -  NA | -  NA  NA  1  -  -  -  -  -  -  -  1  -  -  -  -  -  -  -  NA  NA  NA  NA  -  -  5  -  3 | 1  -  -  1  -  1  -  -  -  -  -  2  -  -  -  1  NA  -  -  -  -  -  -  -  1  9  -  NA | 1  NA  NA  -  1  -  -  -  -  -  -  -  1  -  -  -  -  -  -  NA  NA  NA  NA  1  -  9  -  4 |
