# Supplementary material for: Toxicological and bio-distribution profile of a GM-CSF-expressing, double-targeted, chimeric oncolytic adenovirus ONCOS-102 – Support for clinical studies on advanced cancer treatment
Source: PLoS One. 2017 Aug 10;12(8):e0182715. doi: 10.1371/journal.pone.0182715 (PMC5552138; doi:10.1371/journal.pone.0182715)
Supplement: S5 Table — - organ examined, no pathological findings / organ not examined NA not applicable GRADE 1 minimal/very few/very small GRADE 2 slight/few/small GRADE 3 moderate/moderate number/moderate size GRADE 4 marked/many/large ) finding unilateral in paired organs P finding present, severity not scored (DOCX) [file pone.0182715.s005.docx]

| **Dose group** | **C-TOX** | | **D1-TOX** | | **D2-TOX** | | **D2-TOX CP** | | **D3-TOX** | | **C-TOX-R** | | **D3-TOX-R** | |
| --- | --- | --- | --- | --- | --- | --- | --- | --- | --- | --- | --- | --- | --- | --- |
| **Sex** | **M** | **F** | **M** | **F** | **M** | **F** | **M** | **F** | **M** | **F** | **M** | **F** | **M** | **F** |
| **Number of animals** | **10** | **10** | **10** | **10** | **9** | **8** | **10** | **10** | **10** | **10** | **5** | **5** | **5** | **5** |
| **Organ findings** |  |  |  |  |  |  |  |  |  |  |  |  |  | |
| **Autolysis**  **Abdominal cavity**  -adhesion  **Adrenal gland**  -enlarged  **Dentition**  -missing incisors  **Epididymides**  -enlarged  -reduced  yellow nodule  **Heart**  -hemopericardium  **Kidneys**  -knurled surface  -pale colour  -veostasis  **Liver**  -cyst  -malformation  -enlarged  -marked structure  -pale colour  -pale foci  -prominent nodule  -retracted focus  -venostasis  -yellow focus  **Lungs**  -focal hemorrhage  -venostasis  **Lymph node cervical**  -enlarged  -hemorrhage  **Pericardium**  -adhesions  -whitish colour  **Skin**  -fat-like nodule in subcutis  **Stomach**  -black-brown foci  **Testes**  -adhesion  -flaccid  -reduced  **Throacic cavity**  -hemothorax  **Thymus**  -atrophy  **Urinary bladder**  -dilatation  **Uterus**  -dilatation  -pale nodule | -  -  1  -  -  -  -  -  -  -  -  1  -  -  -  -  -  -  -  -  -  5  -  -  -  -  -  -  -  -  -  -  -  10  -  NA  NA | -  -  -  -  NA  NA  NA  -  -  -  -  -  -  -  -  -  -  -  -  -  -  2  -  -  -  -  -  -  -  NA  NA  NA  -  10  -  8  - | 1  -  1  -  1  1  1  -  -  -  1  -  -  -  -  -  -  -  -  1  -  4  1  -  -  -  -  -  -  1  -  2  -  9  1  NA  NA | -  -  -  -  NA  NA  NA  -  -  -  -  -  -  -  -  -  -  -  -  -  -  4  -  -  -  -  -  -  -  NA  NA  NA  -  10  -  6  - | -  -  -  -  -  -  -  -  -  -  -  -  -  -  -  -  -  -  -  -  -  4  -  -  -  -  -  -  -  1  -  3  -  9  -  NA  NA | 1  -  1  -  NA  NA  NA  1  -  -  -  -  -  -  -  -  -  -  -  -  -  4  -  1  -  -  -  -  -  NA  NA  NA  1  7  -  2  2 | -  1  -  -  -  -  -  -  -  1  -  -  -  -  1  -  1  -  -  -  -  5  -  -  -  -  -  -  -  1  -  -  -  10  -  NA  NA | -  -  -  -  NA  NA  NA  -  -  -  -  -  -  -  -  -  -  -  -  -  -  6  -  -  -  -  -  -  -  NA  NA  NA  -  10  -  2  10 | -  -  -  1  1  -  -  -  1  -  1  -  -  -  -  -  -  -  -  1  -  3  -  -  -  -  -  -  -  1  1  2  -  10  -  NA  - | -  -  -  -  NA  NA  NA  -  -  -  -  -  -  -  -  -  -  -  1  -  1  3  -  -  -  1  2  1  -  NA  NA  NA  -  10  -  5  - | -  -  -  -  -  -  -  -  -  -  -  1  -  -  -  -  -  1  -  -  -  1  -  -  -  -  -  -  -  -  -  -  -  5  -  NA  NA | -  -  -  -  NA  NA  NA  -  -  -  -  -  1  -  -  -  -  -  -  -  1  2  -  -  1  -  -  -  -  NA  NA  NA  -  5  -  -  - | -  -  -  -  -  -  -  -  -  1  -  -  -  1  -  -  -  -  -  -  -  2  -  -  -  -  -  -  -  -  -  -  -  5  -  -  - | -  -  -  -  NA  NA  NA  -  1  -  -  1  -  -  -  1  -  -  -  -  -  1  -  -  -  -  -  -  -  NA  NA  NA  -  5  -  4  - |
