# Supplementary material for: Toxicological and bio-distribution profile of a GM-CSF-expressing, double-targeted, chimeric oncolytic adenovirus ONCOS-102 – Support for clinical studies on advanced cancer treatment
Source: PLoS One. 2017 Aug 10;12(8):e0182715. doi: 10.1371/journal.pone.0182715 (PMC5552138; doi:10.1371/journal.pone.0182715)
Supplement: S6 Table — Statistically significant difference at the 95.0% confidence level is pointed up in boldx Normality test not passed, which tends to invalidate the tests comparing the standard deviations* Statistically significant difference only between means test groups D1-TOX, D2-TOX, D2-TOX CP, D2-TOX SC or D3-TOX versus control group C-TOX** Statistically significant difference only between medians test groups D1-TOX, D2-TOX, D2-TOX CP, D2-TOX SC or D3-TOX versus control group C-TOX*** Statistically significant difference between means and medians test groups D1-TOX, D2-TOX, D2-TOX CP, D2-TOX SC or D3-TOX versus control group C-TOX+ statistically significant difference among standard deviations (F test)# no variablity in one of the columns (DOCX) [file pone.0182715.s006.docx]

| **Male group**  **Ma** | | **Exam. No.:** | **WBC**  **109/l** | | | | | **RBC**  **1012/l** | | | | | **HGB**  **g/l** | | | | | | | | **HCT**  **l/l** | | | | | | | | | **MCV**  **fl** | | | | | | | | | **MCH**  **pg** | | | | | | | | **MCHC**  **g/l** | | | | | | | | | **PLT**  **109/l** | | | | | | | | | **Ebl.**  **‰** | | | | | | | | | | **SN**  **109/l** | | | | | | | | | | **BN**  **109/l** | | | | | | | | | | **EO**  **109/l** | | | | | | | | **BA**  **109/l** | | | | | | | | **LY**  **109/l** | | | | | | | | **MO**  **109/l** | | | | | | | | **PT**  **sec.** | | | | | | | | | **APTT**  **sec.** | | | | | | | |
| --- | --- | --- | --- | --- | --- | --- | --- | --- | --- | --- | --- | --- | --- | --- | --- | --- | --- | --- | --- | --- | --- | --- | --- | --- | --- | --- | --- | --- | --- | --- | --- | --- | --- | --- | --- | --- | --- | --- | --- | --- | --- | --- | --- | --- | --- | --- | --- | --- | --- | --- | --- | --- | --- | --- | --- | --- | --- | --- | --- | --- | --- | --- | --- | --- | --- | --- | --- | --- | --- | --- | --- | --- | --- | --- | --- | --- | --- | --- | --- | --- | --- | --- | --- | --- | --- | --- | --- | --- | --- | --- | --- | --- | --- | --- | --- | --- | --- | --- | --- | --- | --- | --- | --- | --- | --- | --- | --- | --- | --- | --- | --- | --- | --- | --- | --- | --- | --- | --- | --- | --- | --- | --- | --- | --- | --- | --- | --- | --- | --- | --- | --- | --- | --- | --- | --- | --- | --- | --- | --- | --- | --- | --- | --- |
| C-TOX | | D-7  ANOVA p  K-W test p | +  0.672  0.798 | | | | | x  0.317  0.342 | | | | | 0.054  **0.034** | | | | | | | | 0.474  0.679 | | | | | | | | | 0.874  0.671 | | | | | | | | | **0.047**  **0.006** | | | | | | | | **P<0.0001**  **P<0.0001** | | | | | | | | | +  0.148  0.442 | | | | | | | | | -  - | | | | | | | | | | +  0.484  0.577 | | | | | | | | | | x+  **P<0.0001**  **P<0.0001** | | | | | | | | | | x  0.833  0.844 | | | | | | | | 0.461  0.276 | | | | | | | | 0.912  0.884 | | | | | | | | x+  **P<0.0001**  **P<0.0001** | | | | | | | | +  **0.000**  **0.001** | | | | | | | | | +  0.132  0.171 | | | | | | | |
|  |  | N Mean Median SD | 25  7.83  7.60  3.36 | | | | | 25  9.992  10.000  0.405 | | | | | 25  179.0  180.0  8.9 | | | | | | | | 25  0.6139  0.6160  0.0291 | | | | | | | | | 25  60.3  61.0  2.6 | | | | | | | | | 25  17.66  17.80  0.92 | | | | | | | | 25  291.9  292.0  5.7 | | | | | | | | | 25  822.0  813.0  171.0 | | | | | | | | | 25  0.0  0.0  0.0 | | | | | | | | | | 25  2.697  2.501  1.242 | | | | | | | | | | 25  0.070  0.000  0.111 | | | | | | | | | | 25  0.041  0.000  0.066 | | | | | | | | 25  0.000  0.000  0.000 | | | | | | | | 25  5.009  4.752  2.699 | | | | | | | | 25  0.011  0.000  0.033 | | | | | | | | 25  9.17  9.10  0.88 | | | | | | | | | 21  29.43  28.80  6.35 | | | | | | | |
|  |  | D29  ANOVA p  K-W test p | +  0.359  0.866 | | | | | +  0.701  0.814 | | | | | +  0.108  0.068 | | | | | | | | **0.028**  **0.023** | | | | | | | | | x+  0.750  0.105 | | | | | | | | | 0.598  0.186 | | | | | | | | x+  0.625  0.144 | | | | | | | | | 0.420  0.216 | | | | | | | | | -  - | | | | | | | | | | +  **0.001**  **0.011** | | | | | | | | | | +  **0.000**  **0.000** | | | | | | | | | | +  0.632  0.864 | | | | | | | | x  0.667  0.681 | | | | | | | | 0.478  0.356 | | | | | | | | +  0.157  **0.016** | | | | | | | | +  **P<0.0001**  **P<0.0001** | | | | | | | | | 0.487  0.374 | | | | | | | |
|  |  | N Mean Median SD | 25  7.27  6.70  2.92 | | | | | 25  9.477  9.450  0.614 | | | | | 25  174.3  177.0  13.9 | | | | | | | | 25  0.5920  0.5960  0.0403 | | | | | | | | | 25  61.5  62.0  3.6 | | | | | | | | | 25  18.46  18.70  0.86 | | | | | | | | 25  292.9  298.0  16.4 | | | | | | | | | 25  764.6  755.0  260.1 | | | | | | | | | 25  0.0  0.0  0.0 | | | | | | | | | | 25  1.950  1.892  0.771 | | | | | | | | | | 25  0.106  0.067  0.122 | | | | | | | | | | 25  0.070  0.000  0.107 | | | | | | | | 25  0.004  0.000  0.018 | | | | | | | | 25  5.016  4.623  2.154 | | | | | | | | 25  0.121  0.102  0.131 | | | | | | | | 25  8.48  8.40  0.51 | | | | | | | | | 24  27.10  26.95  3.31 | | | | | | | |
|  |  | D190  ANOVA p  K-W test p | 0.969  0.997 | | | | | +  **0.011**  **0.012** | | | | | +  0.146  **0.044** | | | | | | | | 0.058  **0.019** | | | | | | | | | +  0.076  **0.025** | | | | | | | | | +  **0.001**  **0.001** | | | | | | | | **0.001**  **0.003** | | | | | | | | | x  0.077  **0.008** | | | | | | | | | x  0.224  0.223 | | | | | | | | | | +  0.803  0.455 | | | | | | | | | | 0.310  0.332 | | | | | | | | | | 0.826  0.731 | | | | | | | | -  - | | | | | | | | 0.669  0.762 | | | | | | | | 0.844  0.650 | | | | | | | | **P<0.0001**  **0.001** | | | | | | | | | +  **P<0.0001**  **0.000** | | | | | | | |
|  |  | N Mean Median SD | 15  6.67  5.40  3.30 | | | | | 15  8.844  8.840  0.296 | | | | | 15  162.1  164.0  10.0 | | | | | | | | 15  0.5161  0.5230  0.0308 | | | | | | | | | 15  58.1  59.0  2.9 | | | | | | | | | 15  18.15  18.50  1.13 | | | | | | | | 15  311.8  315.0  7.8 | | | | | | | | | 15  407.7  381.0  175.1 | | | | | | | | | 15  0.1  0.0  0.4 | | | | | | | | | | 15  1.848  1.140  1.754 | | | | | | | | | | 15  0.167  0.096  0.200 | | | | | | | | | | 15  0.065  0.000  0.106 | | | | | | | | 15  0.000  0.000  0.000 | | | | | | | | 15  4.038  3.456  1.893 | | | | | | | | 15  0.555  0.388  0.605 | | | | | | | | 15  8.87  8.70  0.83 | | | | | | | | | 15  24.53  24.10  4.70 | | | | | | | |
|  |  | D255  t-test  MW test | 0.243  0.421 | | | | | 0.879  1.000 | | | | | 0.914  0.841 | | | | | | | | 0.975  0.841 | | | | | | | | | 0.915  0.691 | | | | | | | | | 0.870  0.421 | | | | | | | | 0.480  0.421 | | | | | | | | | 0.913  1.000 | | | | | | | | | -  - | | | | | | | | | | 0.434  0.421 | | | | | | | | | | 0.371  0.095 | | | | | | | | | | 0.430  0.430 | | | | | | | | -  - | | | | | | | | 0.275  0.310 | | | | | | | | **0.049**  **0.032** | | | | | | | | 0.163  0.222 | | | | | | | | | 0.737  1.000 | | | | | | | |
|  |  | N Mean Median SD | 5  5.40  4.60  2.45 | | | | | 5  9.932  9.860  0.426 | | | | | 5  168.6  174.0  23.3 | | | | | | | | 5  0.5902  0.6110  0.0813 | | | | | | | | | 5  59.2  62.0  7.5 | | | | | | | | | 5  16.98  17.70  2.19 | | | | | | | | 5  285.8  285.0  4.8 | | | | | | | | | 5  836.2  658.0  457.1 | | | | | | | | | 5  0.2  0.0  0.4 | | | | | | | | | | 5  1.388  1.012  0.964 | | | | | | | | | | 5  0.221  0.123  0.259 | | | | | | | | | | 5  0.038  0.000  0.064 | | | | | | | | 5  0.000  0.000  0.000 | | | | | | | | 5  3.694  3.479  1.252 | | | | | | | | 5  0.060  0.049  0.045 | | | | | | | | 5  10.32  10.30  0.55 | | | | | | | | | 5  25.24  22.10  8.63 | | | | | | | |
| D1-TOX | | D-7  Dunnet’s MC test  Dunn’s MC test | P > 0.05  P > 0.05 | P > 0.05  P > 0.05 | | | | | | | | P > 0.05  P > 0.05 | | | | | | | | | | | P > 0.05  P > 0.05 | | | | | | | | | | P > 0.05  P > 0.05 | | | | | | | | *  **P < 0.05**  P > 0.05 | | | | | | | | ***  **P < 0.01**  **P < 0.001** | | | | | | | | | P > 0.05  P > 0.05 | | | | | | | | | -  - | | | | | | | | | | P > 0.05  P > 0.05 | | | | | | | | | | ***  **P < 0.01**  **P < 0.001** | | | | | | | | | | x  P > 0.05  P > 0.05 | | | | | | | | P > 0.05  P > 0.05 | | | | | | | | P > 0.05  P > 0.05 | | | | | | | | ***  **P < 0.01**  **P < 0.001** | | | | | | | | | | | P > 0.05  P > 0.05 | | | | | | | P > 0.05  P > 0.05 | | | | |
|  |  | N Mean Median SD | 20  10.76  7.70  7.01 | 20  9.994  10.025  0.354 | | | | | | | | 20  185.3  187.5  9.1 | | | | | | | | | | | 20  0.6037  0.6080  0.0314 | | | | | | | | | | 20  59.7  60.0  2.6 | | | | | | | | 20  18.37  18.50  0.88 | | | | | | | | 20  308.2  307.0  5.4 | | | | | | | | | 20  805.6  867.5  221.8 | | | | | | | | | 20  0.0  0.0  0.0 | | | | | | | | | | 20  3.476  2.238  2.702 | | | | | | | | | | 20  0.589  0.420  0.567 | | | | | | | | | | 20  0.039  0.000  0.065 | | | | | | | | 20  0.000  0.000  0.000 | | | | | | | | 20  6.094  5.231  4.016 | | | | | | | | 20  0.556  0.266  0.724 | | | | | | | | | | | 20  9.46  9.35  0.72 | | | | | | | 20  27.74  26.55  3.22 | | | | |
|  |  | D29  Dunnet’s MC test  Dunn’s MC test | P > 0.05  P > 0.05 | P > 0.05  P > 0.05 | | | | | | | | P > 0.05  P > 0.05 | | | | | | | | | | | P > 0.05  P > 0.05 | | | | | | | | | | P > 0.05  P > 0.05 | | | | | | | | P > 0.05  P > 0.05 | | | | | | | | P > 0.05  P > 0.05 | | | | | | | | | P > 0.05  P > 0.05 | | | | | | | | | -  - | | | | | | | | | | P > 0.05  P > 0.05 | | | | | | | | | | P > 0.05  P > 0.05 | | | | | | | | | | x  P > 0.05  P > 0.05 | | | | | | | | x  P > 0.05  P > 0.05 | | | | | | | | P > 0.05  P > 0.05 | | | | | | | | **  P > 0.05  **P < 0.01** | | | | | | | | | | | P > 0.05  P > 0.05 | | | | | | | P > 0.05  P > 0.05 | | | | |
|  |  | N Mean Median SD | 20  7.58  7.10  3.84 | 20  9.598  9.565  0.426 | | | | | | | | 20  173.2  173.0  9.2 | | | | | | | | | | | 20  0.5840  0.5875  0.0381 | | | | | | | | | | 20  61.2  61.0  2.4 | | | | | | | | 20  18.10  18.05  0.69 | | | | | | | | 20  295.5  296.0  4.2 | | | | | | | | | 20  869.3  832.0  211.5 | | | | | | | | | 20  0.0  0.0  0.0 | | | | | | | | | | 20  3.050  2.669  1.651 | | | | | | | | | | 20  0.152  0.103  0.151 | | | | | | | | | | 20  0.055  0.000  0.081 | | | | | | | | 20  0.006  0.000  0.025 | | | | | | | | 20  3.979  3.765  2.359 | | | | | | | | 20  0.334  0.287  0.189 | | | | | | | | | | | 20  9.08  9.10  0.51 | | | | | | | 20  27.25  27.45  3.20 | | | | |
|  |  | D190  Dunnet’s MC test  Dunn’s MC test | P > 0.05  P > 0.05 | ***  **P < 0.05**  **P < 0.05** | | | | | | | | P > 0.05  P > 0.05 | | | | | | | | | | | P > 0.05  P > 0.05 | | | | | | | | | | P > 0.05  P > 0.05 | | | | | | | | P > 0.05  P > 0.05 | | | | | | | | P > 0.05  P > 0.05 | | | | | | | | | P > 0.05  P > 0.05 | | | | | | | | | P > 0.05  P > 0.05 | | | | | | | | | | P > 0.05  P > 0.05 | | | | | | | | | | P > 0.05  P > 0.05 | | | | | | | | | | P > 0.05  P > 0.05 | | | | | | | | -  - | | | | | | | | P > 0.05  P > 0.05 | | | | | | | | P > 0.05  P > 0.05 | | | | | | | | | | | P > 0.05  P > 0.05 | | | | | | | ***  **P < 0.01**  **P < 0.01** | | | | |
|  |  | N Mean Median SD | 9  6.32  5.80  3.44 | 9  8.328  8.380  0.494 | | | | | | | | 9  155.6  158.0  7.9 | | | | | | | | | | | 9  0.4910  0.4810  0.0227 | | | | | | | | | | 9  58.6  58.0  1.2 | | | | | | | | 9  18.72  18.70  0.47 | | | | | | | | 9  319.7  320.0  6.9 | | | | | | | | | 9  378.3  377.0  102.9 | | | | | | | | | 9  0.0  0.0  0.0 | | | | | | | | | | 9  2.024  1.682  0.731 | | | | | | | | | | 9  0.191  0.118  0.179 | | | | | | | | | | 9  0.040  0.000  0.061 | | | | | | | | 9  0.000  0.000  0.000 | | | | | | | | 9  3.465  3.149  2.374 | | | | | | | | 9  0.602  0.414  0.534 | | | | | | | | | | | 9  9.39  9.30  0.82 | | | | | | | 9  17.52  16.90  1.79 | | | | |
| D2-TOX | | D-7  Dunnet’s MC test  Dunn’s MC test | P > 0.05  P > 0.05 | | | P > 0.05  P > 0.05 | | | | | | | | | | | P > 0.05  P > 0.05 | | | | | | | | | P > 0.05  P > 0.05 | | | | | | | | | P > 0.05  P > 0.05 | | | | | | | | P > 0.05  P > 0.05 | | | | | | | | P > 0.05  P > 0.05 | | | | | | | | P > 0.05  P > 0.05 | | | | | | | | | -  - | | | | | | | | | | P > 0.05  P > 0.05 | | | | | | | | | | P > 0.05  P > 0.05 | | | | | | | | | P > 0.05  P > 0.05 | | | | | | | | x  P > 0.05  P > 0.05 | | | | | | | | P > 0.05  P > 0.05 | | | | | | | | **  P > 0.05  **P < 0.01** | | | | | | | | P > 0.05  P > 0.05 | | | | | | | | | | | | P > 0.05  P > 0.05 | | |
|  |  | N Mean Median SD | 20  9.55  8.60  4.53 | | | 20  9.988  10.045  0.425 | | | | | | | | | | | 20  178.5  180.5  7.4 | | | | | | | | | 20  0.6082  0.6140  0.0239 | | | | | | | | | 20  60.3  60.0  2.8 | | | | | | | | 20  17.68  17.75  0.90 | | | | | | | | 20  293.3  294.0  3.3 | | | | | | | | 20  840.6  867.5  178.3 | | | | | | | | | 20  0.0  0.0  0.0 | | | | | | | | | | 20  3.038  2.799  1.179 | | | | | | | | | | 20  0.124  0.142  0.096 | | | | | | | | | 20  0.067  0.015  0.096 | | | | | | | | 20  0.004  0.000  0.017 | | | | | | | | 20  6.167  5.132  3.636 | | | | | | | | 20  0.145  0.104  0.132 | | | | | | | | 20  9.99  9.45  1.83 | | | | | | | | | | | | 20  29.61  29.25  3.10 | | |
|  |  | D29  Dunnet’s MC test  Dunn’s MC test | P > 0.05  P > 0.05 | | | P > 0.05  P > 0.05 | | | | | | | | | | | P > 0.05  P > 0.05 | | | | | | | | | P > 0.05  P > 0.05 | | | | | | | | | P > 0.05  P > 0.05 | | | | | | | | P > 0.05  P > 0.05 | | | | | | | | P > 0.05  P > 0.05 | | | | | | | | P > 0.05  P > 0.05 | | | | | | | | | -  - | | | | | | | | | | *  **P < 0.01**  P > 0.05 | | | | | | | | | | ***  **P < 0.01**  **P < 0.01** | | | | | | | | | x  P > 0.05  P > 0.05 | | | | | | | | P > 0.05  P > 0.05 | | | | | | | | P > 0.05  P > 0.05 | | | | | | | | P > 0.05  P > 0.05 | | | | | | | | ***  **P < 0.01**  **P < 0.001** | | | | | | | | | | | | P > 0.05  P > 0.05 | | |
|  |  | N Mean Median SD | 19  10.41  7.10  7.42 | | | 19  9.397  9.470  0.547 | | | | | | | | | | | 19  169.8  173.0  9.6 | | | | | | | | | 19  0.5674  0.5780  0.0404 | | | | | | | | | 19  61.3  62.0  3.0 | | | | | | | | 19  18.14  18.30  0.85 | | | | | | | | 19  295.6  295.0  4.8 | | | | | | | | 19  835.6  865.0  198.9 | | | | | | | | | 19  0.0  0.0  0.0 | | | | | | | | | | 19  5.217  2.485  4.692 | | | | | | | | | | 19  0.528  0.618  0.478 | | | | | | | | | 19  0.044  0.000  0.086 | | | | | | | | 19  0.000  0.000  0.000 | | | | | | | | 19  4.283  4.260  2.256 | | | | | | | | 19  0.333  0.071  0.492 | | | | | | | | 19  9.92  10.00  0.93 | | | | | | | | | | | | 19  27.65  26.90  4.00 | | |
|  |  | D190  Dunnet’s MC test  Dunn’s MC test | P > 0.05  P > 0.05 | | | P > 0.05  P > 0.05 | | | | | | | | | | | P > 0.05  P > 0.05 | | | | | | | | | P > 0.05  P > 0.05 | | | | | | | | | P > 0.05  P > 0.05 | | | | | | | | P > 0.05  P > 0.05 | | | | | | | | P > 0.05  P > 0.05 | | | | | | | | P > 0.05  P > 0.05 | | | | | | | | | P > 0.05  P > 0.05 | | | | | | | | | | P > 0.05  P > 0.05 | | | | | | | | | | P > 0.05  P > 0.05 | | | | | | | | | P > 0.05  P > 0.05 | | | | | | | | -  - | | | | | | | | P > 0.05  P > 0.05 | | | | | | | | P > 0.05  P > 0.05 | | | | | | | | P > 0.05  P > 0.05 | | | | | | | | | | | | P > 0.05  P > 0.05 | | |
|  |  | N Mean Median SD | 9  5.90  5.80  1.86 | | | 9  8.740  8.850  0.260 | | | | | | | | | | | 9  164.0  164.0  4.6 | | | | | | | | | 9  0.5184  0.5160  0.0211 | | | | | | | | | 9  59.1  59.0  2.0 | | | | | | | | 9  18.86  18.70  0.39 | | | | | | | | 9  319.0  322.0  7.3 | | | | | | | | 9  434.9  457.0  135.3 | | | | | | | | | 9  0.0  0.0  0.0 | | | | | | | | | | 9  1.949  1.540  1.140 | | | | | | | | | | 9  0.144  0.092  0.143 | | | | | | | | | 9  0.053  0.046  0.058 | | | | | | | | 9  0.000  0.000  0.000 | | | | | | | | 9  3.339  2.720  1.363 | | | | | | | | 9  0.414  0.264  0.371 | | | | | | | | 9  8.37  8.30  0.90 | | | | | | | | | | | | 9  25.43  25.70  3.07 | | |
| D2-TOX CP | | D-7  Dunnet’s MC test  Dunn’s MC test | P > 0.05  P > 0.05 | | | | | P > 0.05  P > 0.05 | | | | | | | P > 0.05  P > 0.05 | | | | | | | | | P > 0.05  P > 0.05 | | | | | | | | P > 0.05  P > 0.05 | | | | | | | | | P > 0.05  P > 0.05 | | | | | | | | | P > 0.05  P > 0.05 | | | | | | | | | | | P > 0.05  P > 0.05 | | | | | | | | | -  - | | | | | | | | | | P > 0.05  P > 0.05 | | | | | | | | | | P > 0.05  P > 0.05 | | | | | | | P > 0.05  P > 0.05 | | | | | | | | x  P > 0.05  P > 0.05 | | | | | | | | P > 0.05  P > 0.05 | | | | | | | | **  P > 0.05  **P < 0.01** | | | | | | | | ***  **P < 0.01**  **P < 0.05** | | | | | | | | | | | | P > 0.05  P > 0.05 | | |
|  |  | N Mean Median SD | 20  9.65  7.05  7.78 | | | | | 20  10.012  9.980  0.554 | | | | | | | 20  178.3  180.0  10.4 | | | | | | | | | 20  0.6022  0.6080  0.0372 | | | | | | | | 20  59.4  60.0  2.8 | | | | | | | | | 20  17.57  17.70  0.97 | | | | | | | | | 20  295.1  295.0  4.0 | | | | | | | | | | | 20  774.2  834.0  176.5 | | | | | | | | | 20  0.0  0.0  0.0 | | | | | | | | | | 20  3.633  2.365  3.364 | | | | | | | | | | 20  0.182  0.108  0.226 | | | | | | | 20  0.063  0.023  0.076 | | | | | | | | 20  0.023  0.000  0.076 | | | | | | | | 20  5.560  4.529  4.282 | | | | | | | | 20  0.185  0.090  0.236 | | | | | | | | 20  10.46  10.20  1.65 | | | | | | | | | | | | 20  26.63  26.45  4.11 | | |
|  |  | D29  Dunnet’s MC test  Dunn’s MC test | P > 0.05  P > 0.05 | | | | | P > 0.05  P > 0.05 | | | | | | | P > 0.05  P > 0.05 | | | | | | | | | P > 0.05  P > 0.05 | | | | | | | | P > 0.05  P > 0.05 | | | | | | | | | P > 0.05  P > 0.05 | | | | | | | | | P > 0.05  P > 0.05 | | | | | | | | | | | P > 0.05  P > 0.05 | | | | | | | | | -  - | | | | | | | | | | P > 0.05  P > 0.05 | | | | | | | | | | x  P > 0.05  P > 0.05 | | | | | | | x  P > 0.05  P > 0.05 | | | | | | | | P > 0.05  P > 0.05 | | | | | | | | P > 0.05  P > 0.05 | | | | | | | | x  P > 0.05  P > 0.05 | | | | | | | | ***  **P < 0.01**  **P < 0.001** | | | | | | | | | | | | P > 0.05  P > 0.05 | | |
|  |  | N Mean Median SD | 20  8.16  5.05  4.88 | | | | | 20  9.545  9.530  0.433 | | | | | | | 20  172.8  174.0  6.5 | | | | | | | | | 20  0.5782  0.5815  0.0298 | | | | | | | | 20  61.4  61.0  1.4 | | | | | | | | | 20  18.20  18.20  0.50 | | | | | | | | | 20  297.6  298.0  4.8 | | | | | | | | | | | 20  901.2  879.5  207.6 | | | | | | | | | 20  0.0  0.0  0.0 | | | | | | | | | | 20  3.432  2.176  2.348 | | | | | | | | | | 20  0.205  0.077  0.413 | | | | | | | 20  0.038  0.000  0.066 | | | | | | | | 20  0.000  0.000  0.000 | | | | | | | | 20  4.218  3.183  2.626 | | | | | | | | 20  0.262  0.109  0.299 | | | | | | | | 20  9.71  9.45  1.42 | | | | | | | | | | | | 20  26.09  24.95  4.27 | | |
|  |  | D190  Dunnet’s MC test  Dunn’s MC test | P > 0.05  P > 0.05 | | | | | *  **P < 0.05**  P > 0.05 | | | | | | | P > 0.05  P > 0.05 | | | | | | | | | P > 0.05  P > 0.05 | | | | | | | | ***  **P < 0.05**  **P < 0.05** | | | | | | | | | ***  **P < 0.01**  **P < 0.001** | | | | | | | | | ***  **P < 0.01**  **P < 0.001** | | | | | | | | | | | **  P > 0.05  **P < 0.05** | | | | | | | | | P > 0.05  P > 0.05 | | | | | | | | | | P > 0.05  P > 0.05 | | | | | | | | | | P > 0.05  P > 0.05 | | | | | | | x  P > 0.05  P > 0.05 | | | | | | | | -  - | | | | | | | | P > 0.05  P > 0.05 | | | | | | | | P > 0.05  P > 0.05 | | | | | | | | ***  **P < 0.01**  **P < 0.01** | | | | | | | | | | | | P > 0.05  P > 0.05 | | |
|  |  | N Mean Median SD | 10  6.61  5.65  3.11 | | | | | 10  8.333  8.405  0.641 | | | | | | | 10  161.8  159.5  6.9 | | | | | | | | | 10  0.4930  0.4830  0.0359 | | | | | | | | 10  60.4  60.5  1.2 | | | | | | | | | 10  19.62  19.75  0.53 | | | | | | | | | 10  325.9  328.5  7.0 | | | | | | | | | | | 10  273.1  255.5  74.0 | | | | | | | | | 10  0.0  0.0  0.0 | | | | | | | | | | 10  2.743  1.527  2.451 | | | | | | | | | | 10  0.239  0.131  0.256 | | | | | | | 10  0.029  0.000  0.068 | | | | | | | | 10  0.000  0.000  0.000 | | | | | | | | 10  3.115  3.295  1.022 | | | | | | | | 10  0.485  0.442  0.294 | | | | | | | | 10  10.77  10.60  1.46 | | | | | | | | | | | | 10  24.01  23.15  3.95 | | |
| D2-TOX SC | | D-7  Dunnet’s MC test  Dunn’s MC test | P > 0.05  P > 0.05 | | | | | P > 0.05  P > 0.05 | | | | | | | P > 0.05  P > 0.05 | | | | | | | | | P > 0.05  P > 0.05 | | | | | | | | P > 0.05  P > 0.05 | | | | | | | | | P > 0.05  P > 0.05 | | | | | | | | | *  **P < 0.05**  P > 0.05 | | | | | | | | | | | P > 0.05  P > 0.05 | | | | | | | | | -  - | | | | | | | | | | P > 0.05  P > 0.05 | | | | | | | | | | P > 0.05  P > 0.05 | | | | | | | P > 0.05  P > 0.05 | | | | | | | | P > 0.05  P > 0.05 | | | | | | | | P > 0.05  P > 0.05 | | | | | | | | P > 0.05  P > 0.05 | | | | | | | | P > 0.05  P > 0.05 | | | | | | | | | | | | P > 0.05  P > 0.05 | | |
|  |  | N Mean Median SD | 5  9.42  7.00  5.94 | | | | | 5  9.740  10.100  0.924 | | | | | | | 5  174.8  174.0  8.7 | | | | | | | | | 5  0.5874  0.5880  0.0368 | | | | | | | | 5  60.0  61.0  3.7 | | | | | | | | | 5  17.90  17.90  1.25 | | | | | | | | | 5  298.0  299.0  5.0 | | | | | | | | | | | 5  601.4  446.0  415.6 | | | | | | | | | 5  0.0  0.0  0.0 | | | | | | | | | | 5  4.039  3.640  2.972 | | | | | | | | | | 5  0.087  0.065  0.078 | | | | | | | 5  0.041  0.000  0.062 | | | | | | | | 5  0.014  0.000  0.031 | | | | | | | | 5  5.149  4.290  3.134 | | | | | | | | 5  0.091  0.114  0.091 | | | | | | | | 5  8.68  8.50  1.36 | | | | | | | | | | | | 5  26.90  26.00  3.31 | | |
|  |  | D29  Dunnet’s MC test  Dunn’s MC test | P > 0.05  P > 0.05 | | | | | P > 0.05  P > 0.05 | | | | | | | P > 0.05  P > 0.05 | | | | | | | | | P > 0.05  P > 0.05 | | | | | | | | P > 0.05  P > 0.05 | | | | | | | | | P > 0.05  P > 0.05 | | | | | | | | | P > 0.05  P > 0.05 | | | | | | | | | | | P > 0.05  P > 0.05 | | | | | | | | | -  - | | | | | | | | | | P > 0.05  P > 0.05 | | | | | | | | | | P > 0.05  P > 0.05 | | | | | | | P > 0.05  P > 0.05 | | | | | | | | P > 0.05  P > 0.05 | | | | | | | | P > 0.05  P > 0.05 | | | | | | | | P > 0.05  P > 0.05 | | | | | | | | P > 0.05  P > 0.05 | | | | | | | | | | | | P > 0.05  P > 0.05 | | |
|  |  | N Mean Median SD | 5  8.66  8.70  1.15 | | | | | 5  9.332  9.130  0.959 | | | | | | | 5  163.8  161.0  8.9 | | | | | | | | | 5  0.5568  0.5440  0.0296 | | | | | | | | 5  61.8  61.0  2.2 | | | | | | | | | 5  18.32  17.90  0.68 | | | | | | | | | 5  294.4  294.0  3.6 | | | | | | | | | | | 5  898.4  824.0  202.2 | | | | | | | | | 5  0.0  0.0  0.0 | | | | | | | | | | 5  3.824  3.570  1.853 | | | | | | | | | | 5  0.359  0.420  0.135 | | | | | | | 5  0.090  0.000  0.151 | | | | | | | | 5  0.000  0.000  0.000 | | | | | | | | 5  4.226  3.108  1.678 | | | | | | | | 5  0.162  0.174  0.138 | | | | | | | | 5  8.72  8.70  0.47 | | | | | | | | | | | | 5  25.74  23.50  5.05 | | |
| D3-TOX | | D-7  Dunnet's MC test  Dunn's MC test | P > 0.05  P > 0.05 | | P > 0.05  P > 0.05 | | | | | | | | | | | P > 0.05  P > 0.05 | | | | | | | | | P > 0.05  P > 0.05 | | | | | | | | | P > 0.05  P > 0.05 | | | | | | | | P > 0.05  P > 0.05 | | | | | | | | | | P > 0.05  P > 0.05 | | | | | | | | x  P > 0.05  P > 0.05 | | | | | | | | | -  - | | | | | | | | | | P > 0.05  P > 0.05 | | | | | | | | | | P > 0.05  P > 0.05 | | | | | | | | | x  P > 0.05  P > 0.05 | | | | | | | | x  P > 0.05  P > 0.05 | | | | | | | | P > 0.05  P > 0.05 | | | | | | | | x  P > 0.05  P > 0.05 | | | | | | | | P > 0.05  P > 0.05 | | | | | | | | | | | | P > 0.05  P > 0.05 | |
|  |  | N Mean Median SD | 25  8.46  6.60  5.65 | | 25  8.963  10.200  0.434 | | | | | | | | | | | 25  160.0  179.0  6.8 | | | | | | | | | 25  0.5406  0.6120  0.0239 | | | | | | | | | 25  59.0  61.0  2.4 | | | | | | | | 25  17.77  17.80  0.74 | | | | | | | | | | 25  290.3  293.0  3.9 | | | | | | | | 24  617.8  905.5  298.6 | | | | | | | | | 25  0.0  0.0  0.0 | | | | | | | | | | 25  2.671  2.166  1.779 | | | | | | | | | | 25  0.140  0.172  0.195 | | | | | | | | | 25  0.022  0.000  0.094 | | | | | | | | 25  0.000  0.000  0.050 | | | | | | | | 25  5.179  4.092  4.149 | | | | | | | | 25  0.449  0.043  0.235 | | | | | | | | 25  8.71  8.90  0.85 | | | | | | | | | | | | 24  24.76  26.85  5.15 | |
|  |  | D29  Dunnet's MC test  Dunn's MC test | P > 0.05  P > 0.05 | | P > 0.05  P > 0.05 | | | | | | | | | | | P > 0.05  P > 0.05 | | | | | | | | | P > 0.05  P > 0.05 | | | | | | | | | P > 0.05  P > 0.05 | | | | | | | | P > 0.05  P > 0.05 | | | | | | | | | | P > 0.05  P > 0.05 | | | | | | | | P > 0.05  P > 0.05 | | | | | | | | | -  - | | | | | | | | | | P > 0.05  P > 0.05 | | | | | | | | | | P > 0.05  P > 0.05 | | | | | | | | | x  P > 0.05  P > 0.05 | | | | | | | | P > 0.05  P > 0.05 | | | | | | | | P > 0.05  P > 0.05 | | | | | | | | P > 0.05  P > 0.05 | | | | | | | | ***  **P < 0.01**  **P < 0.001** | | | | | | | | | | | | P > 0.05  P > 0.05 | |
|  |  | N Mean Median SD | 24  8.05  7.70  4.40 | | 24  9.598  9.610  0.361 | | | | | | | | | | | 24  175.9  177.5  6.1 | | | | | | | | | 24  0.5975  0.5960  0.0220 | | | | | | | | | 24  62.3  63.0  2.5 | | | | | | | | 24  18.34  18.45  0.72 | | | | | | | | | | 24  294.4  293.5  3.8 | | | | | | | | 24  847.7  837.0  230.0 | | | | | | | | | 24  0.0  0.0  0.0 | | | | | | | | | | 24  2.170  1.321  1.794 | | | | | | | | | | 24  0.171  0.080  0.270 | | | | | | | | | 24  0.037  0.000  0.064 | | | | | | | | 24  0.000  0.000  0.000 | | | | | | | | 24  5.214  4.763  2.750 | | | | | | | | 24  0.253  0.120  0.316 | | | | | | | | 24  9.56  9.55  0.81 | | | | | | | | | | | | 24  25.69  26.05  3.70 | |
|  |  | D190  Dunnet's MC test  Dunn's MC test | P > 0.05  P > 0.05 | | P > 0.05  P > 0.05 | | | | | | | | | | | P > 0.05  P > 0.05 | | | | | | | | | P > 0.05  P > 0.05 | | | | | | | | | P > 0.05  P > 0.05 | | | | | | | | P > 0.05  P > 0.05 | | | | | | | | | | P > 0.05  P > 0.05 | | | | | | | | P > 0.05  P > 0.05 | | | | | | | | | P > 0.05  P > 0.05 | | | | | | | | | | P > 0.05  P > 0.05 | | | | | | | | | | P > 0.05  P > 0.05 | | | | | | | | | P > 0.05  P > 0.05 | | | | | | | | -  - | | | | | | | | P > 0.05  P > 0.05 | | | | | | | | P > 0.05  P > 0.05 | | | | | | | | P > 0.05  P > 0.05 | | | | | | | | | | | | P > 0.05  P > 0.05 | |
|  |  | N Mean Median SD | 14  6.81  6.00  3.58 | | 14  8.787  8.655  0.455 | | | | | | | | | | | 14  162.1  161.0  4.7 | | | | | | | | | 14  0.5106  0.5095  0.0176 | | | | | | | | | 14  59.1  59.0  1.5 | | | | | | | | 14  18.71  18.85  0.72 | | | | | | | | | | 14  317.0  317.0  7.7 | | | | | | | | 14  357.4  339.0  122.5 | | | | | | | | | 14  0.0  0.0  0.0 | | | | | | | | | | 14  2.184  1.260  2.152 | | | | | | | | | | 14  0.079  0.037  0.119 | | | | | | | | | 14  0.050  0.000  0.069 | | | | | | | | 14  0.000  0.000  0.000 | | | | | | | | 14  3.871  3.819  1.602 | | | | | | | | 14  0.630  0.485  0.461 | | | | | | | | 14  8.77  9.00  0.82 | | | | | | | | | | | | 14  23.51  23.10  2.26 | |
|  |  | D255 |  | |  | | | | | | | | | | |  | | | | | | | | |  | | | | | | | | |  | | | | | | | |  | | | | | | | | | |  | | | | | | | |  | | | | | | | | |  | | | | | | | | | |  | | | | | | | | | |  | | | | | | | | |  | | | | | | | |  | | | | | | | |  | | | | | | | | *** | | | | | | | |  | | | | | | | | | | | |  | |
|  |  | N Mean Median SD | 5  7.74  7.50  3.35 | | 5  9.878  9.920  0.643 | | | | | | | | | | | 5  170.0  173.0  15.4 | | | | | | | | | 5  0.5888  0.6000  0.0516 | | | | | | | | | 5  59.6  58.0  3.2 | | | | | | | | 5  17.16  17.10  0.93 | | | | | | | | | | 5  288.2  288.0  5.4 | | | | | | | | 4  804.3  688.5  370.6 | | | | | | | | | 5  0.0  0.0  0.0 | | | | | | | | | | 5  2.036  1.875  1.474 | | | | | | | | | | 5  0.383  0.234  0.282 | | | | | | | | | 5  0.080  0.072  0.096 | | | | | | | | 5  0.000  0.000  0.000 | | | | | | | | 5  5.074  4.575  2.317 | | | | | | | | 5  0.166  0.144  0.092 | | | | | | | | 5  9.50  9.80  1.06 | | | | | | | | | | | | 5  23.26  22.40  9.38 | |
| **Female group**  **Ma** | | **Exam. No.:** | **WBC**  **109/l** | | | | | | **RBC**  **1012/l** | | | | | **HGB**  **g/l** | | | | | | | | **HCT**  **l/l** | | | | | | | | | **MCV**  **fl** | | | | | | | | | **MCH**  **pg** | | | | | | | | **MCHC**  **g/l** | | | | | | | | | **PLT**  **109/l** | | | | | | | | | **Ebl.**  **‰** | | | | | | | | | | **SN**  **109/l** | | | | | | | | | | **BN**  **109/l** | | | | | | | | | | **EO**  **109/l** | | | | | | | | **BA**  **109/l** | | | | | | | | **LY**  **109/l** | | | | | | | | **MO**  **109/l** | | | | | | | | **PT**  **sec.** | | | | | | | | | **APTT**  **sec.** | | | | | | |
| C-TOX | | D-7  ANOVA p  K-W test p | 0.599  0.615 | | | | | | +  0.676  0.641 | | | | | +  0.508  0.096 | | | | | | | | +  0.328  0.153 | | | | | | | | | +  0.874  0.441 | | | | | | | | | +  0.147  0.076 | | | | | | | | +  **P<0.0001**  **P<0.0001** | | | | | | | | | 0.134  0.126 | | | | | | | | | 0.453  0.447 | | | | | | | | | | 0.068  0.085 | | | | | | | | | | +  **0.000**  **0.001** | | | | | | | | | | x+  **0.046**  **0.034** | | | | | | | | x  0.930  0.888 | | | | | | | | 0.876  0.783 | | | | | | | | x+  0.073  0.097 | | | | | | | | x+  0.761  0.407 | | | | | | | | | +  **0.000**  **0.000** | | | | | | |
|  |  | N Mean Median SD | 25  6.78  7.20  3.71 | | | | | | 25  9.459  9.570  0.552 | | | | | 25  173.8  175.0  11.1 | | | | | | | | 25  0.5954  0.5980  0.0418 | | | | | | | | | 25  62.6  63.0  1.5 | | | | | | | | | 25  18.29  18.40  0.45 | | | | | | | | 25  292.5  293.0  4.3 | | | | | | | | | 25  906.9  961.0  141.4 | | | | | | | | | 25  0.0  0.0  0.0 | | | | | | | | | | 25  2.057  2.295  1.034 | | | | | | | | | | 25  0.138  0.092  0.139 | | | | | | | | | | 25  0.027  0.000  0.055 | | | | | | | | 25  0.004  0.000  0.020 | | | | | | | | 25  4.501  4.464  2.773 | | | | | | | | 25  0.053  0.000  0.081 | | | | | | | | 25  9.60  9.00  2.82 | | | | | | | | | 25  30.59  29.90  4.65 | | | | | | |
|  |  | D29  ANOVA p  K-W test p | 0.118  0.096 | | | | | | 0.926  0.895 | | | | | **0.015**  **0.006** | | | | | | | | 0.450  0.265 | | | | | | | | | x+  0.756  0.606 | | | | | | | | | +  0.057  **0.040** | | | | | | | | +  **0.000**  **0.002** | | | | | | | | | **0.044**  **0.038** | | | | | | | | | -  - | | | | | | | | | | 0.553  0.663 | | | | | | | | | | +  **0.002**  **0.001** | | | | | | | | | | +  0.221  0.437 | | | | | | | | x  0.374  0.368 | | | | | | | | **0.018**  **0.014** | | | | | | | | +  0.227  **0.011** | | | | | | | | 0.109  0.115 | | | | | | | | | **0.020**  **0.047** | | | | | | |
|  |  | N Mean Median SD | 24  6.81  5.85  3.53 | | | | | | 24  9.023  9.070  0.419 | | | | | 24  170.4  169.0  10.0 | | | | | | | | 24  0.5767  0.5755  0.0315 | | | | | | | | | 24  63.8  64.0  1.7 | | | | | | | | | 24  18.88  18.95  0.70 | | | | | | | | 24  295.4  295.0  6.2 | | | | | | | | | 24  911.7  947.5  175.3 | | | | | | | | | 24  0.0  0.0  0.0 | | | | | | | | | | 24  2.135  1.476  1.317 | | | | | | | | | | 24  0.129  0.076  0.159 | | | | | | | | | | 24  0.057  0.038  0.071 | | | | | | | | 24  0.009  0.000  0.021 | | | | | | | | 24  4.339  3.782  2.241 | | | | | | | | 24  0.144  0.129  0.109 | | | | | | | | 24  8.86  8.75  0.80 | | | | | | | | | 24  25.78  25.20  2.74 | | | | | | |
|  |  | D190  ANOVA p  K-W test p | +  0.089  **0.042** | | | | | | x+  0.756  0.907 | | | | | x+  0.779  0.713 | | | | | | | | +  0.338  0.054 | | | | | | | | | +  **0.015**  **0.006** | | | | | | | | | +  **0.001**  **0.003** | | | | | | | | **0.028**  0.124 | | | | | | | | | 0.423  0.460 | | | | | | | | | x  0.630  0.615 | | | | | | | | | | +  0.536  0.436 | | | | | | | | | | +  0.628  0.260 | | | | | | | | | | +  0.940  0.824 | | | | | | | | x  0.240  0.223 | | | | | | | | +  **0.025**  **0.004** | | | | | | | | +  0.345  0.541 | | | | | | | | +  0.204  **0.046** | | | | | | | | | 0.278  0.344 | | | | | | |
|  |  | N Mean Median SD | 15  6.41  5.50  3.31 | | | | | | 15  8.141  8.290  0.777 | | | | | 15  154.7  157.0  15.1 | | | | | | | | 15  0.4815  0.4960  0.0462 | | | | | | | | | 15  59.5  60.0  2.2 | | | | | | | | | 15  18.76  18.90  0.89 | | | | | | | | 15  315.8  316.0  5.0 | | | | | | | | | 15  458.6  475.0  156.5 | | | | | | | | | 15  0.1  0.0  0.3 | | | | | | | | | | 15  2.141  1.802  1.439 | | | | | | | | | | 15  0.163  0.075  0.218 | | | | | | | | | | 15  0.060  0.000  0.095 | | | | | | | | 15  0.012  0.000  0.033 | | | | | | | | 15  3.595  3.658  1.774 | | | | | | | | 15  0.435  0.371  0.329 | | | | | | | | 15  8.97  9.10  0.85 | | | | | | | | | 15  24.35  23.90  3.51 | | | | | | |
|  |  | D255  t-test  MW test | 0.516  0.548 | | | | | | 0.103  0.095 | | | | | **0.028**  **0.032** | | | | | | | | **0.002**  **0.008** | | | | | | | | | 0.651  0.548 | | | | | | | | | 0.839  0.691 | | | | | | | | **0.024**  0.056 | | | | | | | | | 0.936  1.000 | | | | | | | | | -  - | | | | | | | | | | 0.706  0.691 | | | | | | | | | | 0.673  0.310 | | | | | | | | | | 0.680  0.841 | | | | | | | | -  - | | | | | | | | 0.392  0.421 | | | | | | | | 0.338  0.222 | | | | | | | | 0.129  0.095 | | | | | | | | | 0.809  0.421 | | | | | | |
|  |  | N Mean Median SD | 5  7.04  8.10  3.14 | | | | | | 5  9.734  9.450  0.734 | | | | | 5  169.2  170.0  5.6 | | | | | | | | 5  0.6002  0.6010  0.0139 | | | | | | | | | 5  62.2  64.0  5.3 | | | | | | | | | 5  17.52  18.20  1.67 | | | | | | | | 5  282.2  282.0  5.6 | | | | | | | | | 5  834.0  808.0  261.4 | | | | | | | | | 5  0.0  0.0  0.0 | | | | | | | | | | 5  1.872  1.620  1.500 | | | | | | | | | | 5  0.147  0.152  0.055 | | | | | | | | | | 5  0.181  0.038  0.244 | | | | | | | | 5  0.000  0.000  0.000 | | | | | | | | 5  4.544  3.870  2.103 | | | | | | | | 5  0.296  0.324  0.177 | | | | | | | | 5  11.10  10.40  1.49 | | | | | | | | | 5  28.22  26.00  6.72 | | | | | | |
| D1-TOX | | D-7  Dunnet’s MC test  Dunn’s MC test | P > 0.05  P > 0.05 | | | | P > 0.05  P > 0.05 | | | | | | | | | | | P > 0.05  P > 0.05 | | | | | | | | | P > 0.05  P > 0.05 | | | | | | | | | x  P > 0.05  P > 0.05 | | | | | | | | P > 0.05  P > 0.05 | | | | | | | | | ***  **P < 0.01**  **P < 0.01** | | | | | | | | | P > 0.05  P > 0.05 | | | | | | | | | | x  P > 0.05  P > 0.05 | | | | | | | | | | P > 0.05  P > 0.05 | | | | | | | | | | P > 0.05  P > 0.05 | | | | | | | | | P > 0.05  P > 0.05 | | | | | | | | x  P > 0.05  P > 0.05 | | | | | | | | P > 0.05  P > 0.05 | | | | | | | | P > 0.05  P > 0.05 | | | | | | | | | P > 0.05  P > 0.05 | | | | | | | | P > 0.05  P > 0.05 | |
|  |  | N Mean Median SD | 20  6.56  5.85  3.10 | | | | 20  9.169  9.325  0.956 | | | | | | | | | | | 20  172.7  174.5  12.2 | | | | | | | | | 20  0.5687  0.5855  0.0600 | | | | | | | | | 20  62.1  62.0  1.7 | | | | | | | | 20  18.49  18.40  0.52 | | | | | | | | | 20  297.7  298.5  6.1 | | | | | | | | | 20  924.4  924.5  189.8 | | | | | | | | | | 20  0.1  0.0  0.2 | | | | | | | | | | 20  2.417  2.022  1.452 | | | | | | | | | | 20  0.166  0.152  0.118 | | | | | | | | | 20  0.078  0.040  0.106 | | | | | | | | 20  0.007  0.000  0.024 | | | | | | | | 20  3.745  3.039  2.385 | | | | | | | | 20  0.147  0.080  0.161 | | | | | | | | | 20  9.32  9.10  1.27 | | | | | | | | 20  30.26  29.40  3.87 | |
|  | | D29  Dunnet’s MC test  Dunn’s MC test | P > 0.05  P > 0.05 | | | | P > 0.05  P > 0.05 | | | | | | | | | | | P > 0.05  P > 0.05 | | | | | | | | | P > 0.05  P > 0.05 | | | | | | | | | P > 0.05  P > 0.05 | | | | | | | | P > 0.05  P > 0.05 | | | | | | | | | P > 0.05  P > 0.05 | | | | | | | | | P > 0.05  P > 0.05 | | | | | | | | | | -  - | | | | | | | | | | P > 0.05  P > 0.05 | | | | | | | | | | P > 0.05  P > 0.05 | | | | | | | | | x  P > 0.05  P > 0.05 | | | | | | | | P > 0.05  P > 0.05 | | | | | | | | x*  **P < 0.05**  P > 0.05 | | | | | | | | P > 0.05  P > 0.05 | | | | | | | | | P > 0.05  P > 0.05 | | | | | | | | P > 0.05  P > 0.05 | |
|  |  | N Mean Median SD | 20  4.69  3.15  2.78 | | | | 20  9.059  9.145  0.470 | | | | | | | | | | | 20  171.0  172.0  8.3 | | | | | | | | | 20  0.5795  0.5820  0.0296 | | | | | | | | | 20  63.9  64.0  1.4 | | | | | | | | 20  18.84  18.90  0.41 | | | | | | | | | 20  294.8  295.0  3.3 | | | | | | | | | 20  821.5  819.5  117.3 | | | | | | | | | | 20  0.0  0.0  0.0 | | | | | | | | | | 20  1.909  1.550  1.006 | | | | | | | | | | 20  0.055  0.037  0.057 | | | | | | | | | 20  0.029  0.000  0.046 | | | | | | | | 20  0.000  0.000  0.000 | | | | | | | | 20  2.576  1.726  1.860 | | | | | | | | 20  0.122  0.074  0.133 | | | | | | | | | 20  9.67  9.60  0.97 | | | | | | | | 20  27.98  27.10  2.85 | |
|  |  | D190  Dunnet’s MC test  Dunn’s MC test | P > 0.05  P > 0.05 | | | | P > 0.05  P > 0.05 | | | | | | | | | | | P > 0.05  P > 0.05 | | | | | | | | | P > 0.05  P > 0.05 | | | | | | | | | P > 0.05  P > 0.05 | | | | | | | | P > 0.05  P > 0.05 | | | | | | | | | P > 0.05  P > 0.05 | | | | | | | | | P > 0.05  P > 0.05 | | | | | | | | | | P > 0.05  P > 0.05 | | | | | | | | | | P > 0.05  P > 0.05 | | | | | | | | | | P > 0.05  P > 0.05 | | | | | | | | | P > 0.05  P > 0.05 | | | | | | | | P > 0.05  P > 0.05 | | | | | | | | P > 0.05  P > 0.05 | | | | | | | | P > 0.05  P > 0.05 | | | | | | | | | P > 0.05  P > 0.05 | | | | | | | | P > 0.05  P > 0.05 | |
|  |  | N Mean Median SD | 10  4.83  4.80  1.20 | | | | 10  8.318  8.325  0.217 | | | | | | | | | | | 10  159.3  158.0  4.1 | | | | | | | | | 10  0.4969  0.4945  0.0206 | | | | | | | | | 10  60.7  60.5  0.8 | | | | | | | | 10  19.33  19.25  0.27 | | | | | | | | | 10  318.0  318.0  7.4 | | | | | | | | | 10  438.8  451.5  75.8 | | | | | | | | | | 10  0.0  0.0  0.0 | | | | | | | | | | 10  1.502  1.394  0.558 | | | | | | | | | | 10  0.131  0.123  0.069 | | | | | | | | | 10  0.040  0.000  0.058 | | | | | | | | 10  0.000  0.000  0.000 | | | | | | | | 10  2.823  2.657  1.093 | | | | | | | | 10  0.302  0.342  0.094 | | | | | | | | | 10  8.79  8.55  0.54 | | | | | | | | 10  22.05  22.10  2.75 | |
| D2-TOX | | D-7  Dunnet’s MC test  Dunn’s MC test | P > 0.05  P > 0.05 | | | | P > 0.05  P > 0.05 | | | | | | | | | | | P > 0.05  P > 0.05 | | | | | | | | | P > 0.05  P > 0.05 | | | | | | | | | P > 0.05  P > 0.05 | | | | | | | | P > 0.05  P > 0.05 | | | | | | | | | P > 0.05  P > 0.05 | | | | | | | | | P > 0.05  P > 0.05 | | | | | | | | | P > 0.05  P > 0.05 | | | | | | | | | | P > 0.05  P > 0.05 | | | | | | | | | | P > 0.05  P > 0.05 | | | | | | | | | x  P > 0.05  P > 0.05 | | | | | | | | x  P > 0.05  P > 0.05 | | | | | | | | P > 0.05  P > 0.05 | | | | | | | | P > 0.05  P > 0.05 | | | | | | | | | P > 0.05  P > 0.05 | | | | | | | ***  **P < 0.01**  **P < 0.01** | | | |
|  |  | N Mean Median SD | 20  7.63  7.70  3.04 | | | | 20  9.348  9.430  0.583 | | | | | | | | | | | 20  168.2  170.0  10.9 | | | | | | | | | 20  0.5814  0.5875  0.0386 | | | | | | | | | 20  62.3  63.0  1.5 | | | | | | | | 20  18.02  18.00  0.52 | | | | | | | | | 20  289.5  289.5  5.0 | | | | | | | | | 20  930.7  912.5  156.7 | | | | | | | | | 20  0.0  0.0  0.0 | | | | | | | | | | 20  2.816  2.428  1.255 | | | | | | | | | | 20  0.084  0.000  0.127 | | | | | | | | | 20  0.019  0.000  0.039 | | | | | | | | 20  0.004  0.000  0.017 | | | | | | | | 20  4.627  4.858  1.907 | | | | | | | | 20  0.074  0.073  0.074 | | | | | | | | | 20  10.27  9.65  2.36 | | | | | | | 20  25.49  25.90  2.46 | | | |
|  |  | D29  Dunnet’s MC test  Dunn’s MC test | P > 0.05  P > 0.05 | | | | P > 0.05  P > 0.05 | | | | | | | | | | | P > 0.05  P > 0.05 | | | | | | | | | P > 0.05  P > 0.05 | | | | | | | | | P > 0.05  P > 0.05 | | | | | | | | P > 0.05  P > 0.05 | | | | | | | | | P > 0.05  P > 0.05 | | | | | | | | | P > 0.05  P > 0.05 | | | | | | | | | -  - | | | | | | | | | | P > 0.05  P > 0.05 | | | | | | | | | | P > 0.05  P > 0.05 | | | | | | | | | P > 0.05  P > 0.05 | | | | | | | | x  P > 0.05  P > 0.05 | | | | | | | | P > 0.05  P > 0.05 | | | | | | | | **  P > 0.05  **P < 0.01** | | | | | | | | | P > 0.05  P > 0.05 | | | | | | | P > 0.05  P > 0.05 | | | |
|  |  | N Mean Median SD | 16  5.30  4.50  2.36 | | | | 16  9.083  9.120  0.303 | | | | | | | | | | | 16  172.6  171.5  6.2 | | | | | | | | | 16  0.5837  0.5810  0.0198 | | | | | | | | | 16  64.3  64.0  1.0 | | | | | | | | 16  19.01  18.90  0.34 | | | | | | | | | 16  296.2  296.0  2.8 | | | | | | | | | 16  949.4  930.5  142.1 | | | | | | | | | 14  0.0  0.0  0.0 | | | | | | | | | | 16  1.919  1.465  1.122 | | | | | | | | | | 16  0.229  0.159  0.207 | | | | | | | | | 16  0.062  0.045  0.075 | | | | | | | | 16  0.003  0.000  0.012 | | | | | | | | 16  3.043  2.917  1.518 | | | | | | | | 16  0.044  0.017  0.068 | | | | | | | | | 16  9.29  9.35  0.80 | | | | | | | 16  24.52  24.85  3.26 | | | |
|  |  | D190  Dunnet’s MC test  Dunn’s MC test | P > 0.05  P > 0.05 | | | | P > 0.05  P > 0.05 | | | | | | | | | | | P > 0.05  P > 0.05 | | | | | | | | | P > 0.05  P > 0.05 | | | | | | | | | P > 0.05  P > 0.05 | | | | | | | | P > 0.05  P > 0.05 | | | | | | | | | P > 0.05  P > 0.05 | | | | | | | | | P > 0.05  P > 0.05 | | | | | | | | | P > 0.05  P > 0.05 | | | | | | | | | | P > 0.05  P > 0.05 | | | | | | | | | | P > 0.05  P > 0.05 | | | | | | | | | P > 0.05  P > 0.05 | | | | | | | | P > 0.05  P > 0.05 | | | | | | | | P > 0.05  P > 0.05 | | | | | | | | P > 0.05  P > 0.05 | | | | | | | | | P > 0.05  P > 0.05 | | | | | | | P > 0.05  P > 0.05 | | | |
|  |  | N Mean Median SD | 7  4.33  3.90  1.81 | | | | 7  8.294  8.290  0.042 | | | | | | | | | | | 7  157.7  158.0  1.7 | | | | | | | | | 7  0.4904  0.4920  0.0069 | | | | | | | | | 7  61.0  61.0  0.8 | | | | | | | | 7  19.36  19.20  0.47 | | | | | | | | | 7  319.1  320.0  3.9 | | | | | | | | | 7  401.4  400.0  79.8 | | | | | | | | | 5  0.0  0.0  0.0 | | | | | | | | | | 7  1.768  1.794  0.867 | | | | | | | | | | 7  0.072  0.030  0.133 | | | | | | | | | 7  0.041  0.000  0.110 | | | | | | | | 7  0.000  0.000  0.000 | | | | | | | | 7  2.086  1.950  0.673 | | | | | | | | 7  0.361  0.290  0.187 | | | | | | | | | 7  8.43  7.80  1.76 | | | | | | | 7  22.90  23.90  2.70 | | | |
| D2-TOX CP | | D-7  Dunnet’s MC test  Dunn’s MC test | P > 0.05  P > 0.05 | | | | | | | | P > 0.05  P > 0.05 | | | | | | | | | P > 0.05  P > 0.05 | | | | | | | | P > 0.05  P > 0.05 | | | | | | | | | P > 0.05  P > 0.05 | | | | | | | | P > 0.05  P > 0.05 | | | | | | | | | ***  **P < 0.01**  **P < 0.05** | | | | | | | | | P > 0.05  P > 0.05 | | | | | | | | | | P > 0.05  P > 0.05 | | | | | | | | | | P > 0.05  P > 0.05 | | | | | | | | | | P > 0.05  P > 0.05 | | | | | | P > 0.05  P > 0.05 | | | | | | | | x  P > 0.05  P > 0.05 | | | | | | | | P > 0.05  P > 0.05 | | | | | | | | P > 0.05  P > 0.05 | | | | | | | | P > 0.05  P > 0.05 | | | | | | | *  **P < 0.01**  P > 0.05 | | | | | |
|  |  | N Mean Median SD | 20  6.73  6.40  3.02 | | | | | | | | 20  9.187  9.335  0.676 | | | | | | | | | 20  170.5  172.0  14.7 | | | | | | | | 20  0.5725  0.5825  0.0504 | | | | | | | | | 20  62.3  62.0  3.0 | | | | | | | | 20  18.50  18.40  0.82 | | | | | | | | | 20  297.1  297.0  3.0 | | | | | | | | | 20  966.9  984.5  193.9 | | | | | | | | | | 20  0.0  0.0  0.0 | | | | | | | | | | 20  2.327  2.030  1.320 | | | | | | | | | | 20  0.099  0.069  0.111 | | | | | | 20  0.080  0.037  0.118 | | | | | | | | 20  0.003  0.000  0.009 | | | | | | | | 20  4.138  3.812  1.931 | | | | | | | | 20  0.082  0.067  0.098 | | | | | | | | 20  9.67  9.40  1.52 | | | | | | | 20  26.77  26.95  3.71 | | | | | |
|  |  | D29  Dunnet’s MC test  Dunn’s MC test | P > 0.05  P > 0.05 | | | | | | | | P > 0.05  P > 0.05 | | | | | | | | | P > 0.05  P > 0.05 | | | | | | | | P > 0.05  P > 0.05 | | | | | | | | | P > 0.05  P > 0.05 | | | | | | | | P > 0.05  P > 0.05 | | | | | | | | | P > 0.05  P > 0.05 | | | | | | | | | P > 0.05  P > 0.05 | | | | | | | | | | -  - | | | | | | | | | | P > 0.05  P > 0.05 | | | | | | | | | | P > 0.05  P > 0.05 | | | | | | P > 0.05  P > 0.05 | | | | | | | | x  P > 0.05  P > 0.05 | | | | | | | | ***  **P < 0.05**  **P < 0.05** | | | | | | | | P > 0.05  P > 0.05 | | | | | | | | P > 0.05  P > 0.05 | | | | | | | P > 0.05  P > 0.05 | | | | | |
|  |  | N Mean Median SD | 19  4.40  3.70  2.65 | | | | | | | | 19  8.978  8.930  0.461 | | | | | | | | | 19  170.3  171.0  5.8 | | | | | | | | 19  0.5772  0.5780  0.0208 | | | | | | | | | 19  64.3  64.0  2.5 | | | | | | | | 19  18.87  19.00  0.86 | | | | | | | | | 19  294.5  294.0  3.0 | | | | | | | | | 19  892.1  896.0  138.3 | | | | | | | | | | 19  0.0  0.0  0.0 | | | | | | | | | | 19  1.687  1.404  0.886 | | | | | | | | | | 19  0.056  0.048  0.062 | | | | | | 19  0.034  0.024  0.052 | | | | | | | | 19  0.001  0.000  0.006 | | | | | | | | 19  2.484  1.952  1.725 | | | | | | | | 19  0.138  0.072  0.190 | | | | | | | | 19  9.12  9.20  1.05 | | | | | | | 19  25.04  23.50  4.75 | | | | | |
|  |  | D190  Dunnet’s MC test  Dunn’s MC test | P > 0.05  P > 0.05 | | | | | | | | P > 0.05  P > 0.05 | | | | | | | | | P > 0.05  P > 0.05 | | | | | | | | P > 0.05  P > 0.05 | | | | | | | | | ***  **P < 0.01**  **P < 0.01** | | | | | | | | ***  **P < 0.01**  **P < 0.01** | | | | | | | | | *  **P < 0.05**  P > 0.05 | | | | | | | | | P > 0.05  P > 0.05 | | | | | | | | | | P > 0.05  P > 0.05 | | | | | | | | | | P > 0.05  P > 0.05 | | | | | | | | | | P > 0.05  P > 0.05 | | | | | | P > 0.05  P > 0.05 | | | | | | | | P > 0.05  P > 0.05 | | | | | | | | ***  **P < 0.05**  **P < 0.05** | | | | | | | | P > 0.05  P > 0.05 | | | | | | | | P > 0.05  P > 0.05 | | | | | | | P > 0.05  P > 0.05 | | | | | |
|  |  | N Mean Median SD | 10  3.81  3.20  1.77 | | | | | | | | 10  8.091  8.380  0.527 | | | | | | | | | 10  156.5  156.5  2.3 | | | | | | | | 10  0.4746  0.4750  0.0128 | | | | | | | | | 10  61.7  62.0  1.9 | | | | | | | | 10  20.03  20.25  0.90 | | | | | | | | | 10  324.6  321.0  9.2 | | | | | | | | | 10  433.3  440.0  117.1 | | | | | | | | | | 10  0.0  0.0  0.0 | | | | | | | | | | 10  1.484  1.057  1.200 | | | | | | | | | | 10  0.081  0.040  0.126 | | | | | | 10  0.036  0.026  0.042 | | | | | | | | 10  0.000  0.000  0.000 | | | | | | | | 10  1.844  1.762  0.480 | | | | | | | | 10  0.359  0.294  0.210 | | | | | | | | 10  8.31  8.35  0.63 | | | | | | | 10  24.22  24.05  2.66 | | | | | |
| D2-TOX SC | | D-7  Dunnet’s MC test  Dunn’s MC test | P > 0.05  P > 0.05 | | | | | | | | P > 0.05  P > 0.05 | | | | | | | | | P > 0.05  P > 0.05 | | | | | | | | P > 0.05  P > 0.05 | | | | | | | | | P > 0.05  P > 0.05 | | | | | | | | P > 0.05  P > 0.05 | | | | | | | | | P > 0.05  P > 0.05 | | | | | | | | | P > 0.05  P > 0.05 | | | | | | | | | | P > 0.05  P > 0.05 | | | | | | | | | | P > 0.05  P > 0.05 | | | | | | | | | | P > 0.05  P > 0.05 | | | | | | P > 0.05  P > 0.05 | | | | | | | | P > 0.05  P > 0.05 | | | | | | | | P > 0.05  P > 0.05 | | | | | | | | P > 0.05  P > 0.05 | | | | | | | | P > 0.05  P > 0.05 | | | | | | | P > 0.05  P > 0.05 | | | | | |
|  |  | N Mean Median SD | 5  8.16  6.60  4.16 | | | | | | | | 5  9.474  9.420  0.263 | | | | | | | | | 5  176.0  177.0  2.3 | | | | | | | | 5  0.5976  0.5940  0.0082 | | | | | | | | | 5  63.2  63.0  1.5 | | | | | | | | 5  18.56  18.40  0.52 | | | | | | | | | 5  294.2  294.0  2.3 | | | | | | | | | 5  821.8  741.0  226.4 | | | | | | | | | | 5  0.0  0.0  0.0 | | | | | | | | | | 5  3.371  2.772  1.988 | | | | | | | | | | 5  0.241  0.198  0.180 | | | | | | 5  0.096  0.120  0.096 | | | | | | | | 5  0.000  0.000  0.000 | | | | | | | | 5  4.350  3.564  2.035 | | | | | | | | 5  0.102  0.066  0.104 | | | | | | | | 5  9.14  9.30  0.29 | | | | | | | 5  30.44  30.60  0.85 | | | | | |
|  |  | D29  Dunnet’s MC test  Dunn’s MC test | P > 0.05  P > 0.05 | | | | | | | | P > 0.05  P > 0.05 | | | | | | | | | P > 0.05  P > 0.05 | | | | | | | | P > 0.05  P > 0.05 | | | | | | | | | P > 0.05  P > 0.05 | | | | | | | | P > 0.05  P > 0.05 | | | | | | | | | P > 0.05  P > 0.05 | | | | | | | | | P > 0.05  P > 0.05 | | | | | | | | | | -  - | | | | | | | | | | P > 0.05  P > 0.05 | | | | | | | | | | P > 0.05  P > 0.05 | | | | | | P > 0.05  P > 0.05 | | | | | | | | P > 0.05  P > 0.05 | | | | | | | | P > 0.05  P > 0.05 | | | | | | | | P > 0.05  P > 0.05 | | | | | | | | P > 0.05  P > 0.05 | | | | | | | P > 0.05  P > 0.05 | | | | | |
|  |  | N Mean Median SD | 5  4.62  3.90  2.66 | | | | | | | | 5  9.040  8.970  0.522 | | | | | | | | | 5  172.6  175.0  6.8 | | | | | | | | 5  0.5818  0.5940  0.0325 | | | | | | | | | 5  64.4  64.0  1.1 | | | | | | | | 5  18.96  19.00  0.38 | | | | | | | | | 5  294.4  294.0  2.6 | | | | | | | | | 5  769.4  781.0  220.8 | | | | | | | | | | 5  0.0  0.0  0.0 | | | | | | | | | | 5  1.716  2.028  1.146 | | | | | | | | | | 5  0.197  0.184  0.155 | | | | | | 5  0.013  0.000  0.018 | | | | | | | | 5  0.000  0.000  0.000 | | | | | | | | 5  2.656  1.700  2.094 | | | | | | | | 5  0.038  0.031  0.034 | | | | | | | | 5  9.44  9.40  0.50 | | | | | | | 5  25.50  25.50  2.82 | | | | | |
| D3-TOX | D-7  Dunnet's MC test  Dunn's MC test | P > 0.05  P > 0.05 | | | | | | | P > 0.05  P > 0.05 | | | | | | | | | P > 0.05  P > 0.05 | | | | | | | | | | P > 0.05  P > 0.05 | | | | | | | | | P > 0.05  P > 0.05 | | | | | | | | P > 0.05  P > 0.05 | | | | | | | | | P > 0.05  P > 0.05 | | | | | | | | | P > 0.05  P > 0.05 | | | | | | | | | | P > 0.05  P > 0.05 | | | | | | | | | | P > 0.05  P > 0.05 | | | | | | | | | | *  **P < 0.01**  P > 0.05 | | | | | | | | P > 0.05  P > 0.05 | | | | | | | | x  P > 0.05  P > 0.05 | | | | | | | | P > 0.05  P > 0.05 | | | | | | | | P > 0.05  P > 0.05 | | | | | | | | | P > 0.05  P > 0.05 | | | | | | | | P > 0.05  P > 0.05 |  |
|  | N Mean Median SD | 25  8.07  7.50  3.99 | | | | | | | 25  9.272  9.390  0.640 | | | | | | | | | 25  169.8  172.0  9.4 | | | | | | | | | | 25  0.5766  0.5830  0.0337 | | | | | | | | | 25  62.2  62.0  2.7 | | | | | | | | 25  18.36  18.30  0.77 | | | | | | | | | 25  294.7  293.0  4.8 | | | | | | | | | 25  1 023.0  1 016.0  210.5 | | | | | | | | | | 25  0.0  0.0  0.0 | | | | | | | | | | 25  3.154  2.688  1.735 | | | | | | | | | | 25  0.311  0.232  0.284 | | | | | | | | 25  0.090  0.056  0.118 | | | | | | | | 25  0.002  0.000  0.010 | | | | | | | | 25  4.388  4.125  2.597 | | | | | | | | 25  0.128  0.084  0.140 | | | | | | | | | 25  9.69  9.20  1.97 | | | | | | | | 22  29.97  31.50  5.12 |  |
|  | D29  Dunnet's MC test  Dunn's MC test | P > 0.05  P > 0.05 | | | | | | | P > 0.05  P > 0.05 | | | | | | | | | *  **P < 0.05**  P > 0.05 | | | | | | | | | | P > 0.05  P > 0.05 | | | | | | | | | P > 0.05  P > 0.05 | | | | | | | | P > 0.05  P > 0.05 | | | | | | | | | ***  **P < 0.01**  **P < 0.01** | | | | | | | | | P > 0.05  P > 0.05 | | | | | | | | | | -  - | | | | | | | | | | P > 0.05  P > 0.05 | | | | | | | | | | P > 0.05  P > 0.05 | | | | | | | | P > 0.05  P > 0.05 | | | | | | | | x  P > 0.05  P > 0.05 | | | | | | | | P > 0.05  P > 0.05 | | | | | | | | P > 0.05  P > 0.05 | | | | | | | | | P > 0.05  P > 0.05 | | | | | | | | P > 0.05  P > 0.05 |  |
|  | N Mean Median SD | 24  5.87  4.70  3.54 | | | | | | | 24  8.944  8.955  0.470 | | | | | | | | | 24  164.3  164.5  7.7 | | | | | | | | | | 24  0.5669  0.5640  0.0254 | | | | | | | | | 24  63.5  64.0  2.9 | | | | | | | | 24  18.36  18.60  0.98 | | | | | | | | | 24  289.9  290.5  5.6 | | | | | | | | | 24  944.1  907.0  180.5 | | | | | | | | | | 24  0.0  0.0  0.0 | | | | | | | | | | 24  1.571  1.270  0.893 | | | | | | | | | | 24  0.140  0.089  0.149 | | | | | | | | 24  0.081  0.008  0.130 | | | | | | | | 24  0.003  0.000  0.014 | | | | | | | | 24  3.922  3.450  2.541 | | | | | | | | 24  0.154  0.068  0.224 | | | | | | | | | 24  9.48  9.25  1.19 | | | | | | | | 24  27.09  27.15  3.18 |  |
|  | D190  Dunnet's MC test  Dunn's MC test | P > 0.05  P > 0.05 | | | | | | | P > 0.05  P > 0.05 | | | | | | | | | P > 0.05  P > 0.05 | | | | | | | | | | P > 0.05  P > 0.05 | | | | | | | | | P > 0.05  P > 0.05 | | | | | | | | P > 0.05  P > 0.05 | | | | | | | | | P > 0.05  P > 0.05 | | | | | | | | | P > 0.05  P > 0.05 | | | | | | | | | | P > 0.05  P > 0.05 | | | | | | | | | | P > 0.05  P > 0.05 | | | | | | | | | | P > 0.05  P > 0.05 | | | | | | | | x  P > 0.05  P > 0.05 | | | | | | | | P > 0.05  P > 0.05 | | | | | | | | P > 0.05  P > 0.05 | | | | | | | | P > 0.05  P > 0.05 | | | | | | | | | P > 0.05  P > 0.05 | | | | | | | | P > 0.05  P > 0.05 |  |
|  | N Mean Median SD | 15  5.98  4.90  3.03 | | | | | | | 15  8.291  8.310  0.336 | | | | | | | | | 15  156.5  157.0  6.9 | | | | | | | | | | 15  0.4936  0.4950  0.0208 | | | | | | | | | 15  59.9  60.0  1.3 | | | | | | | | 15  19.03  19.10  0.64 | | | | | | | | | 15  315.7  318.0  8.0 | | | | | | | | | 15  505.3  482.0  147.6 | | | | | | | | | | 15  0.0  0.0  0.0 | | | | | | | | | | 15  1.776  1.470  0.844 | | | | | | | | | | 15  0.102  0.066  0.146 | | | | | | | | 15  0.042  0.000  0.061 | | | | | | | | 15  0.000  0.000  0.000 | | | | | | | | 15  3.540  2.976  2.153 | | | | | | | | 15  0.519  0.429  0.352 | | | | | | | | | 15  9.15  9.10  0.94 | | | | | | | | 15  24.92  25.00  4.27 |  |
|  | D255 |  | | | | | | |  | | | | | | | | | *** | | | | | | | | | | *** | | | | | | | | |  | | | | | | | |  | | | | | | | | | * | | | | | | | | |  | | | | | | | | | |  | | | | | | | | | |  | | | | | | | | | |  | | | | | | | |  | | | | | | | |  | | | | | | | |  | | | | | | | |  | | | | | | | | |  | | | | | | | |  |  |
|  | N Mean Median SD | 5  8.94  5.90  5.41 | | | | | | | 5  9.008  8.780  0.490 | | | | | | | | | 5  156.2  158.0  9.3 | | | | | | | | | | 5  0.5350  0.5350  0.0290 | | | | | | | | | 5  60.8  62.0  4.1 | | | | | | | | 5  17.72  18.30  1.33 | | | | | | | | | 5  291.4  289.0  4.8 | | | | | | | | | 5  852.8  789.0  434.7 | | | | | | | | | | 5  0.0  0.0  0.0 | | | | | | | | | | 5  2.271  1.357  1.718 | | | | | | | | | | 5  0.214  0.082  0.337 | | | | | | | | 5  0.128  0.059  0.138 | | | | | | | | 5  0.000  0.000  0.000 | | | | | | | | 5  6.157  4.602  3.387 | | | | | | | | 5  0.170  0.059  0.213 | | | | | | | | | 5  9.88  9.60  0.61 | | | | | | | | 5  27.16  23.30  6.67 |  |
