# Supplementary material for: Toxicological and bio-distribution profile of a GM-CSF-expressing, double-targeted, chimeric oncolytic adenovirus ONCOS-102 – Support for clinical studies on advanced cancer treatment
Source: PLoS One. 2017 Aug 10;12(8):e0182715. doi: 10.1371/journal.pone.0182715 (PMC5552138; doi:10.1371/journal.pone.0182715)
Supplement: S7 Table — Statistically significant difference at the 95.0% confidence level is pointed up in boldx Normality test not passed, which tends to invalidate the tests comparing the standard deviations* Statistically significant difference only between means test groups D1-TOX, D2-TOX, D2-TOX CP, D2-TOX SC or D3-TOX versus control group C-TOX** Statistically significant difference only between medians test groups D1-TOX, D2-TOX, D2-TOX CP, D2- TOX SC or D3-TOX versus control group C-TOX*** Statistically significant difference between means and medians test groups D1-TOX, D2-TOX, D2-TOX CP, D2-TOX SC or D3-TOX versus control group C-TOX+ statistically significant difference among standard deviations (F test)# no variablity in one of the columns (DOCX) [file pone.0182715.s007.docx]

| **Male group** | **Exam. No.:** | **LDH**  **µkat/l** | **ALT**  **µkat/l** | **AST**  **µkat/l** | **TP**  **g/l** | **Alb**  **g/l** | **Glo**  **g/l** | **Alb/glo**  **ratio** |
| --- | --- | --- | --- | --- | --- | --- | --- | --- |
| C-TOX | D-7  ANOVA p  K-W test p | +  0.619  0.641 | x+  0.831  0.858 | 0.750  0.848 | +  0.480  0.555 | 0.500  0.210 | 0.815  0.341 | x  0.620  **0.032** |
|  | N Mean Median SD | 25  2.428  2.200  0.826 | 25  1.004  0.870  0.503 | 25  0.758  0.710  0.248 | 25  66.9  66.0  5.3 | 25  27.2  28.0  3.4 | 25  39.8  39.0  5.6 | 25  0.697  0.743  0.124 |
|  | D29  ANOVA p  K-W test p | +  0.150  0.227 | x+  0.744  0.263 | +  0.900  0.443 | +  **0.002**  **0.000** | x  **0.001**  **P<0.0001** | 0.639  0.239 | +  0.869  0.111 |
|  | N Mean Median SD | 17  2.432  2.380  0.622 | 17  1.272  0.940  0.914 | 17  0.918  0.770  0.573 | 17  70.2  68.0  5.2 | 17  28.4  29.0  3.8 | 17  41.8  40.0  6.8 | 17  0.699  0.744  0.142 |
|  | D190  ANOVA p  K-W test p | +  0.269  0.234 | +  0.159  0.500 | +  0.372  0.155 | 0.122  0.081 | 0.564  0.396 | 0.575  0.136 | 0.660  0.154 |
|  | N Mean Median SD | 15  2.009  1.800  0.548 | 15  0.973  0.720  0.554 | 15  0.625  0.630  0.141 | 15  63.2  62.0  5.1 | 15  25.7  26.0  2.6 | 15  37.5  37.0  5.9 | 15  0.701  0.757  0.124 |
|  | D255  t-test  MW test | +  **0.036**  **0.008** | 0.290  0.421 | 0.204  0.095 | **0.029**  0.056 | 0.568  0.841 | 0.116  0.095 | 0.197  0.151 |
|  | N Mean Median SD | 5  2.076  2.120  0.227 | 5  1.374  1.310  0.757 | 5  0.646  0.480  0.405 | 5  64.6  65.0  6.1 | 5  23.8  26.0  5.1 | 5  40.8  37.0  10.0 | 5  0.624  0.703  0.208 |
| D1-TOX | D-7  Dunnet's MC test  Dunn's MC test | P > 0.05  P > 0.05 | P > 0.05  P > 0.05 | P > 0.05  P > 0.05 | P > 0.05  P > 0.05 | P > 0.05  P > 0.05 | P > 0.05  P > 0.05 | P > 0.05  P > 0.05 |
|  | N Mean Median SD | 20  2.243  2.205  0.483 | 20  1.150  0.915  0.579 | 20  0.722  0.720  0.177 | 20  67.8  68.0  2.8 | 20  28.3  29.0  3.2 | 20  39.5  38.5  4.8 | 20  0.733  0.784  0.141 |
|  | D29  Dunnet's MC test  Dunn's MC test | P > 0.05  P > 0.05 | P > 0.05  P > 0.05 | P > 0.05  P > 0.05 | P > 0.05  P > 0.05 | P > 0.05  P > 0.05 | P > 0.05  P > 0.05 | P > 0.05  P > 0.05 |
|  | N Mean Median SD | 20  3.001  2.780  1.100 | 20  1.291  1.020  0.833 | 20  0.908  0.810  0.299 | 20  67.7  68.0  4.7 | 20  25.8  27.0  4.3 | 20  41.9  40.5  7.0 | 20  0.640  0.711  0.174 |
|  | D190  Dunnet's MC test  Dunn's MC test | P > 0.05  P > 0.05 | P > 0.05  P > 0.05 | x  P > 0.05  P > 0.05 | P > 0.05  P > 0.05 | P > 0.05  P > 0.05 | P > 0.05  P > 0.05 | P > 0.05  P > 0.05 |
|  | N Mean Median SD | 9  3.564  2.260  4.054 | 9  0.872  0.720  0.419 | 9  1.110  0.660  1.259 | 9  62.0  61.0  3.5 | 9  24.1  26.0  4.5 | 9  37.9  36.0  7.2 | 9  0.664  0.703  0.172 |
| D2-TOX | D-7  Dunnet‘s MC test  Dunn‘s MC test | P > 0.05  P > 0.05 | P > 0.05  P > 0.05 | P > 0.05  P > 0.05 | P > 0.05  P > 0.05 | P > 0.05  P > 0.05 | P > 0.05  P > 0.05 | P > 0.05  P > 0.05 |
|  | N Mean Median SD | 20  2.136  2.170  0.352 | 20  1.210  1.020  0.618 | 20  0.699  0.650  0.228 | 20  68.8  68.5  4.0 | 20  27.3  28.5  3.8 | 20  41.5  39.5  5.1 | 20  0.673  0.725  0.134 |
|  | D29  Dunnet‘s MC test  Dunn‘s MC test | P > 0.05  P > 0.05 | P > 0.05  P > 0.05 | P > 0.05  P > 0.05 | P > 0.05  P > 0.05 | ***  **P < 0.01**  **P < 0.01** | P > 0.05  P > 0.05 | x  P > 0.05  P > 0.05 |
|  | N Mean Median SD | 17  2.555  2.420  0.606 | 17  1.184  0.850  0.916 | 17  0.835  0.720  0.373 | 17  65.2  67.0  9.7 | 17  23.8  23.0  4.3 | 17  41.5  44.0  10.3 | 17  0.678  0.578  0.480 |
|  | D190  Dunnet‘s MC test  Dunn‘s MC test | P > 0.05  P > 0.05 | P > 0.05  P > 0.05 | P > 0.05  P > 0.05 | P > 0.05  P > 0.05 | P > 0.05  P > 0.05 | P > 0.05  P > 0.05 | P > 0.05  P > 0.05 |
|  | N Mean Median SD | 9  2.292  2.170  0.556 | 9  1.143  1.030  0.715 | 9  0.839  0.770  0.359 | 9  65.8  66.0  6.4 | 9  24.4  25.0  3.4 | 9  41.3  40.0  8.7 | 9  0.617  0.651  0.140 |
| D2-TOX CP | D-7  Dunnet‘s MC test  Dunn‘s MC test | P > 0.05  P > 0.05 | x  P > 0.05  P > 0.05 | P > 0.05  P > 0.05 | P > 0.05  P > 0.05 | P > 0.05  P > 0.05 | P > 0.05  P > 0.05 | x  P > 0.05  P > 0.05 |
|  | N Mean Median SD | 20  2.326  2.135  0.611 | 20  1.023  0.845  0.481 | 20  0.720  0.635  0.218 | 20  66.7  67.5  4.8 | 20  27.7  29.0  3.8 | 20  39.0  37.0  6.1 | 20  0.732  0.775  0.151 |
|  | D29  Dunnet‘s MC test  Dunn‘s MC test | P > 0.05  P > 0.05 | P > 0.05  P > 0.05 | P > 0.05  P > 0.05 | ***  **P < 0.01**  **P < 0.01** | ***  **P < 0.01**  **P < 0.01** | P > 0.05  P > 0.05 | P > 0.05  P > 0.05 |
|  | N Mean Median SD | 20  3.199  2.530  1.806 | 20  1.023  0.810  0.700 | 20  0.862  0.740  0.309 | 20  63.9  63.5  3.6 | 20  24.3  25.5  3.6 | 20  39.6  37.5  4.9 | 20  0.629  0.702  0.142 |
|  | D190  Dunnet‘s MC test  Dunn‘s MC test | P > 0.05  P > 0.05 | P > 0.05  P > 0.05 | P > 0.05  P > 0.05 | P > 0.05  P > 0.05 | P > 0.05  P > 0.05 | P > 0.05  P > 0.05 | P > 0.05  P > 0.05 |
|  | N Mean Median SD | 10  3.046  2.835  1.588 | 10  1.201  0.820  0.979 | 10  0.804  0.725  0.261 | 10  64.5  65.0  5.4 | 10  26.0  26.5  3.5 | 10  38.5  36.0  8.1 | 10  0.705  0.769  0.163 |
| D2-TOX SC | D-7  Dunnet‘s MC test  Dunn‘s MC test | P > 0.05  P > 0.05 | P > 0.05  P > 0.05 | P > 0.05  P > 0.05 | P > 0.05  P > 0.05 | P > 0.05  P > 0.05 | P > 0.05  P > 0.05 | P > 0.05  P > 0.05 |
|  | N Mean Median SD | 5  2.498  2.560  0.535 | 5  1.146  0.690  0.901 | 5  0.842  0.740  0.226 | 5  69.4  69.0  3.2 | 5  28.4  29.0  4.3 | 5  41.0  38.0  5.4 | 5  0.709  0.763  0.163 |
|  | D29  Dunnet‘s MC test  Dunn‘s MC test | P > 0.05  P > 0.05 | P > 0.05  P > 0.05 | P > 0.05  P > 0.05 | P > 0.05  P > 0.05 | P > 0.05  P > 0.05 | P > 0.05  P > 0.05 | P > 0.05  P > 0.05 |
|  | N Mean Median SD | 5  2.526  2.740  0.426 | 5  1.468  0.610  1.770 | 5  0.822  0.690  0.417 | 5  72.2  72.0  2.6 | 5  26.4  26.0  4.2 | 5  45.8  47.0  5.9 | 5  0.593  0.553  0.165 |
| D3-TOX | D-7  Dunnet's MC test  Dunn's MC test | P > 0.05  P > 0.05 | x  P > 0.05  P > 0.05 | P > 0.05  P > 0.05 | P > 0.05  P > 0.05 | P > 0.05  P > 0.05 | P > 0.05  P > 0.05 | P > 0.05  P > 0.05 |
|  | N Mean Median SD | 25  2.315  2.370  0.508 | 25  1.205  0.890  0.874 | 25  0.774  0.740  0.250 | 25  66.2  67.0  6.5 | 25  26.2  27.0  3.7 | 25  40.0  38.0  7.1 | 25  0.678  0.725  0.146 |
|  | D29  Dunnet's MC test  Dunn's MC test | P > 0.05  P > 0.05 | x  P > 0.05  P > 0.05 | P > 0.05  P > 0.05 | P > 0.05  P > 0.05 | P > 0.05  P > 0.05 | P > 0.05  P > 0.05 | P > 0.05  P > 0.05 |
|  | N Mean Median SD | 23  2.533  2.500  0.504 | 23  1.526  1.040  1.448 | 23  0.790  0.710  0.323 | 23  69.5  69.0  4.8 | 21  28.0  29.0  2.8 | 21  41.3  40.0  6.3 | 21  0.696  0.744  0.125 |
|  | D190  Dunnet's MC test  Dunn's MC test | P > 0.05  P > 0.05 | P > 0.05  P > 0.05 | P > 0.05  P > 0.05 | P > 0.05  P > 0.05 | P > 0.05  P > 0.05 | P > 0.05  P > 0.05 | P > 0.05  P > 0.05 |
|  | N Mean Median SD | 14  2.365  2.310  0.688 | 14  1.820  1.340  1.643 | 14  0.811  0.715  0.318 | 14  67.1  66.5  4.2 | 14  26.1  27.0  3.5 | 14  40.9  40.0  6.1 | 14  0.659  0.711  0.152 |
|  | D255 | *** |  |  | * |  |  |  |
| **Female group** | **Exam. No.:** | **LDH**  **µkat/l** | **ALT**  **µkat/l** | **AST**  **µkat/l** | **TP**  **g/l** | **Alb**  **g/l** | **Glo**  **g/l** | **Alb/glo**  **ratio** |
|  | N Mean Median SD | 5  3.168  3.030  0.941 | 5  2.176  1.860  1.390 | 5  1.088  0.830  0.589 | 5  72.8  72.0  3.3 | 5  21.8  20.0  5.5 | 5  51.0  52.0  8.2 | 5  0.450  0.385  0.181 |
| C-TOX | D-7  ANOVA p  K-W test p | 0.922  0.919 | +  0.068  0.209 | 0.532  0.830 | 0.115  0.111 | **0.011**  **0.008** | +  0.175  0.191 | +  0.055  **0.019** |
|  | N Mean Median SD | 25  2.505  2.300  0.706 | 25  0.862  0.740  0.466 | 25  0.846  0.830  0.269 | 25  65.5  66.0  4.3 | 25  24.1  24.0  2.3 | 25  41.4  40.0  3.3 | 25  0.586  0.590  0.069 |
|  | D29  ANOVA p  K-W test p | **0.019**  **0.012** | x  **0.015**  **0.027** | **0.015**  **0.045** | 0.081  0.159 | 0.221  0.210 | 0.066  0.284 | 0.125  0.291 |
|  | N Mean Median SD | 18  2.938  2.730  0.993 | 18  1.061  0.875  0.760 | 18  0.887  0.785  0.283 | 18  68.7  69.0  3.5 | 18  25.8  26.0  2.8 | 18  42.9  42.0  4.0 | 18  0.607  0.619  0.096 |
|  | D190  ANOVA p  K-W test p | +  0.117  0.072 | +  0.089  0.056 | +  0.255  0.207 | +  **0.007**  **P<0.0001** | 0.107  0.107 | +  0.188  **0.004** | +  0.367  0.072 |
|  | N Mean Median SD | 15  2.573  2.560  0.628 | 14  1.302  0.750  1.066 | 15  0.903  0.750  0.456 | 15  70.0  69.0  5.0 | 14  24.1  25.5  3.2 | 14  45.8  43.0  7.7 | 14  0.547  0.602  0.141 |
|  | D255  t-test  MW test | 0.993  1.000 | 0.719  0.691 | 0.869  0.548 | 0.849  0.841 | 0.632  0.691 | 0.895  1.000 | 0.742  0.548 |
|  | N Mean Median SD | 5  2.696  2.800  0.729 | 5  1.996  0.800  2.063 | 5  1.002  0.640  0.727 | 5  69.6  68.0  6.1 | 5  22.4  23.0  3.0 | 5  47.2  43.0  7.4 | 5  0.488  0.558  0.120 |
| D1-TOX | D-7  Dunnet's MC test  Dunn's MC test | P > 0.05  P > 0.05 | P > 0.05  P > 0.05 | P > 0.05  P > 0.05 | P > 0.05  P > 0.05 | *  **P < 0.05**  P > 0.05 | P > 0.05  P > 0.05 | **  P > 0.05  **P < 0.05** |
|  | N Mean Median SD | 20  2.497  2.305  0.836 | 20  0.738  0.690  0.257 | 20  0.799  0.705  0.227 | 20  66.0  65.0  3.4 | 20  25.9  26.0  1.5 | 20  40.1  39.5  2.8 | 20  0.648  0.622  0.052 |
|  | D29  Dunnet's MC test  Dunn's MC test | P > 0.05  P > 0.05 | P > 0.05  P > 0.05 | P > 0.05  P > 0.05 | P > 0.05  P > 0.05 | P > 0.05  P > 0.05 | P > 0.05  P > 0.05 | P > 0.05  P > 0.05 |
|  | N Mean Median SD | 20  3.034  2.975  0.577 | 20  0.911  0.810  0.400 | 20  0.844  0.800  0.202 | 20  66.2  66.5  3.0 | 20  25.4  25.0  1.4 | 20  40.8  40.0  2.7 | 20  0.626  0.615  0.056 |
|  | D190  Dunnet's MC test  Dunn's MC test | P > 0.05  P > 0.05 | P > 0.05  P > 0.05 | P > 0.05  P > 0.05 | ***  **P < 0.05**  **P < 0.001** | P > 0.05  P > 0.05 | **  P > 0.05  **P < 0.05** | P > 0.05  P > 0.05 |
|  | N Mean Median SD | 10  2.216  2.135  0.640 | 10  0.600  0.585  0.128 | 10  0.617  0.665  0.169 | 10  60.3  61.0  4.5 | 10  22.1  23.0  1.4 | 10  38.2  38.0  3.4 | 10  0.580  0.580  0.032 |
| D2-TOX | D-7  Dunnet‘s MC test  Dunn‘s MC test | P > 0.05  P > 0.05 | x  P > 0.05  P > 0.05 | P > 0.05  P > 0.05 | P > 0.05  P > 0.05 | P > 0.05  P > 0.05 | P > 0.05  P > 0.05 | P > 0.05  P > 0.05 |
|  | N Mean Median SD | 20  2.375  2.190  0.493 | 20  0.784  0.685  0.507 | 20  0.792  0.735  0.219 | 20  67.8  68.0  5.3 | 20  24.6  24.5  1.8 | 20  43.3  43.0  5.1 | 20  0.575  0.578  0.072 |
|  | D29  Dunnet‘s MC test  Dunn‘s MC test | *  **P < 0.05**  P > 0.05 | P > 0.05  P > 0.05 | P > 0.05  P > 0.05 | P > 0.05  P > 0.05 | P > 0.05  P > 0.05 | P > 0.05  P > 0.05 | P > 0.05  P > 0.05 |
|  | N Mean Median SD | 14  2.177  2.100  0.652 | 14  0.783  0.810  0.123 | 14  0.745  0.715  0.185 | 14  67.1  67.5  2.5 | 14  25.4  25.0  1.0 | 14  41.8  41.5  2.4 | 14  0.609  0.607  0.042 |
|  | D190  Dunnet‘s MC test  Dunn‘s MC test | P > 0.05  P > 0.05 | P > 0.05  P > 0.05 | P > 0.05  P > 0.05 | P > 0.05  P > 0.05 | P > 0.05  P > 0.05 | P > 0.05  P > 0.05 | P > 0.05  P > 0.05 |
|  | N Mean Median SD | 7  2.214  2.170  0.134 | 7  0.667  0.640  0.122 | 7  0.804  0.770  0.122 | 7  62.9  62.0  2.7 | 7  23.9  24.0  1.6 | 7  39.0  38.0  2.2 | 7  0.613  0.590  0.052 |
| D2-TOX CP | D-7  Dunnet‘s MC test  Dunn‘s MC test | P > 0.05  P > 0.05 | x  P > 0.05  P > 0.05 | P > 0.05  P > 0.05 | P > 0.05  P > 0.05 | P > 0.05  P > 0.05 | P > 0.05  P > 0.05 | P > 0.05  P > 0.05 |
|  | N Mean Median SD | 19  2.529  2.490  0.715 | 19  0.974  0.730  0.744 | 20  0.812  0.820  0.286 | 19  65.9  66.0  4.0 | 18  24.4  25.5  2.4 | 17  41.4  41.0  3.8 | 17  0.596  0.610  0.085 |
|  | D29  Dunnet‘s MC test  Dunn‘s MC test | P > 0.05  P > 0.05 | P > 0.05  P > 0.05 | P > 0.05  P > 0.05 | P > 0.05  P > 0.05 | x  P > 0.05  P > 0.05 | P > 0.05  P > 0.05 | P > 0.05  P > 0.05 |
|  | N Mean Median SD | 19  3.052  3.050  1.002 | 19  1.099  0.900  0.450 | 19  0.998  0.880  0.281 | 19  66.9  66.0  3.4 | 19  24.2  25.0  3.2 | 19  42.7  41.0  4.1 | 19  0.575  0.585  0.109 |
|  | D190  Dunnet‘s MC test  Dunn‘s MC test | P > 0.05  P > 0.05 | P > 0.05  P > 0.05 | P > 0.05  P > 0.05 | ***  **P < 0.05**  **P < 0.001** | P > 0.05  P > 0.05 | **  P > 0.05  **P < 0.01** | P > 0.05  P > 0.05 |
|  | N Mean Median SD | 10  2.427  2.220  0.613 | 10  0.740  0.670  0.333 | 10  0.755  0.705  0.210 | 10  60.5  60.0  3.1 | 10  22.1  22.5  2.7 | 10  38.4  37.5  5.4 | 10  0.590  0.613  0.117 |
| D2-TOX SC | D-7  Dunnet‘s MC test  Dunn‘s MC test | P > 0.05  P > 0.05 | P > 0.05  P > 0.05 | P > 0.05  P > 0.05 | P > 0.05  P > 0.05 | *  **P < 0.05**  P > 0.05 | P > 0.05  P > 0.05 | P > 0.05  P > 0.05 |
|  | N Mean Median SD | 5  2.224  2.140  0.482 | 5  0.766  0.720  0.216 | 5  0.788  0.860  0.152 | 5  71.4  73.0  3.8 | 5  27.0  27.0  1.6 | 5  44.4  46.0  4.6 | 5  0.615  0.609  0.092 |
|  | D29  Dunnet‘s MC test  Dunn‘s MC test | P > 0.05  P > 0.05 | P > 0.05  P > 0.05 | P > 0.05  P > 0.05 | P > 0.05  P > 0.05 | P > 0.05  P > 0.05 | P > 0.05  P > 0.05 | P > 0.05  P > 0.05 |
|  | N Mean Median SD | 4  2.568  2.475  0.876 | 4  0.783  0.780  0.121 | 4  0.770  0.815  0.198 | 4  67.3  68.5  3.6 | 4  26.8  26.5  1.0 | 4  40.5  41.0  3.4 | 4  0.664  0.671  0.059 |
| D3-TOX | D-7  Dunnet's MC test  Dunn's MC test | P > 0.05  P > 0.05 | P > 0.05  P > 0.05 | P > 0.05  P > 0.05 | P > 0.05  P > 0.05 | P > 0.05  P > 0.05 | P > 0.05  P > 0.05 | P > 0.05  P > 0.05 |
|  | N Mean Median SD | 25  2.438  2.420  0.512 | 23  1.264  0.810  0.920 | 25  0.934  0.810  0.376 | 25  66.5  66.0  5.5 | 25  24.0  25.0  2.7 | 25  42.5  41.0  5.9 | 25  0.579  0.610  0.111 |
|  | D29  Dunnet's MC test  Dunn's MC test | P > 0.05  P > 0.05 | x  P > 0.05  P > 0.05 | P > 0.05  P > 0.05 | P > 0.05  P > 0.05 | P > 0.05  P > 0.05 | P > 0.05  P > 0.05 | P > 0.05  P > 0.05 |
|  | N Mean Median SD | 24  2.712  2.760  0.525 | 24  1.570  0.995  1.180 | 24  1.058  0.925  0.388 | 24  69.1  68.0  4.5 | 24  24.7  25.0  2.6 | 24  44.4  42.0  5.4 | 24  0.568  0.605  0.104 |
|  | D190  Dunnet's MC test  Dunn's MC test | P > 0.05  P > 0.05 | P > 0.05  P > 0.05 | P > 0.05  P > 0.05 | x***  **P < 0.01**  **P < 0.05** | P > 0.05  P > 0.05 | x  P > 0.05  P > 0.05 | x  P > 0.05  P > 0.05 |
|  | N Mean Median SD | 15  2.057  1.970  0.432 | 15  1.170  0.760  0.921 | 15  0.851  0.720  0.334 | 15  60.7  64.0  13.2 | 15  21.7  22.0  3.0 | 15  38.9  41.0  15.0 | 15  0.326  0.535  0.752 |
|  | D255 |  |  |  |  |  |  |  |
|  | N Mean Median SD | 5  2.700  2.560  0.746 | 5  1.610  1.100  1.054 | 5  1.070  0.840  0.520 | 5  69.0  69.0  3.1 | 5  21.2  23.0  4.4 | 5  47.8  48.0  6.4 | 5  0.459  0.500  0.148 |
