# Supplementary figures and images for: Toxicological and bio-distribution profile of a GM-CSF-expressing, double-targeted, chimeric oncolytic adenovirus ONCOS-102 – Support for clinical studies on advanced cancer treatment
Source: PLoS One. 2017 Aug 10;12(8):e0182715. doi: 10.1371/journal.pone.0182715 (PMC5552138; doi:10.1371/journal.pone.0182715)

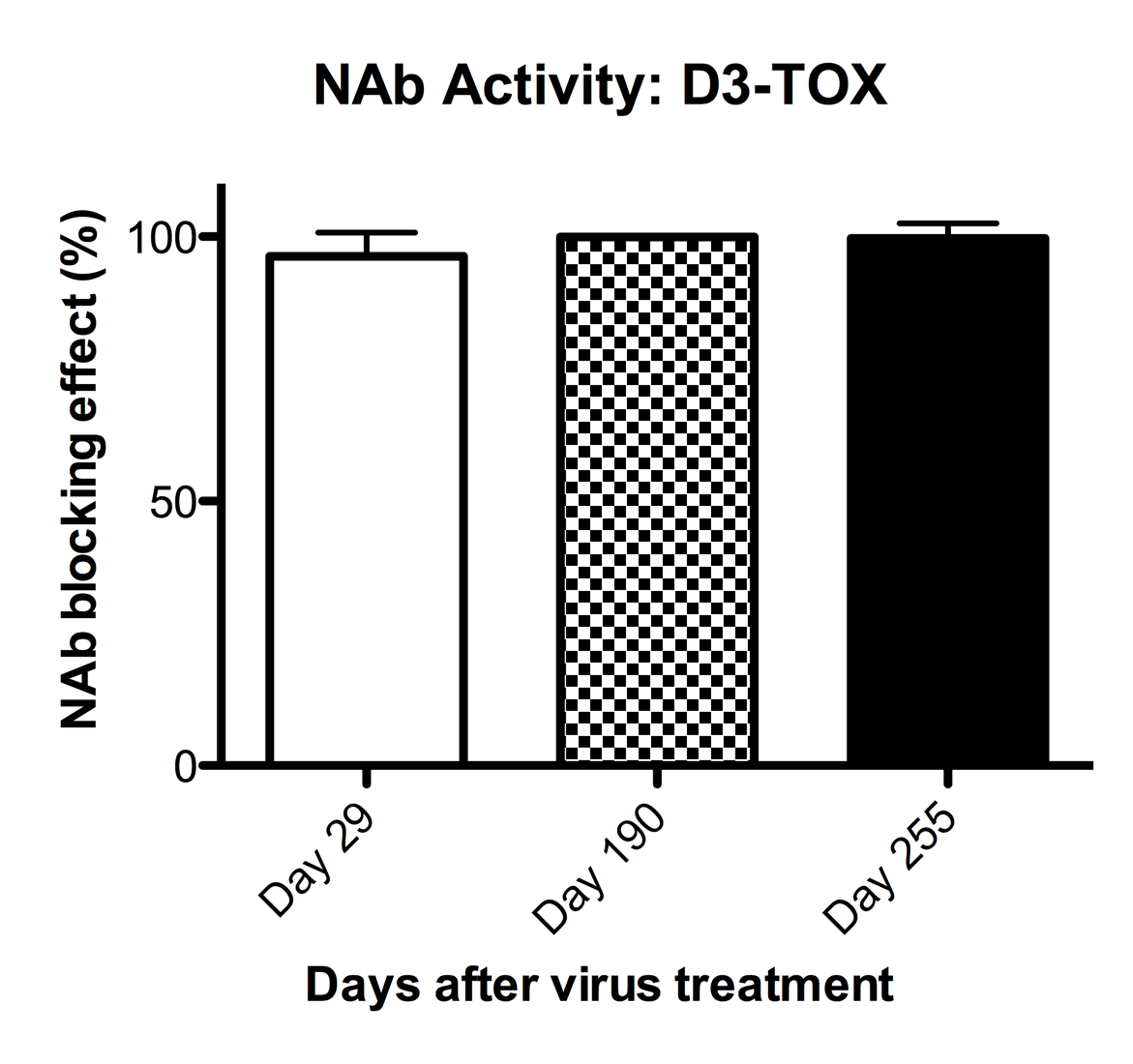

Supplement: S1 Fig — The lowest, medium and highest dose of virus treatment escalated from 1 to 100-fold (D1-TOX 1x; D2-TOX 10x and D3-TOX 100x). Bars present NAb blocking effect ± SEM. Statistical analyses was performed with LSD post hoc comparisons test. Horizontal lines indicate statistically significant differences between sampling days within a group. Stars indicate statistical significance between treatment groups D1-TOX vs. D2-TOX/D3-TOX (*** = P≤ 0.001; ** = P≤ 0.01 and * = P≤ 0.05). There was no statistically significant difference between D2-TOX and D3-TOX groups either sampling days. (TIFF) [file pone.0182715.s008.tiff]

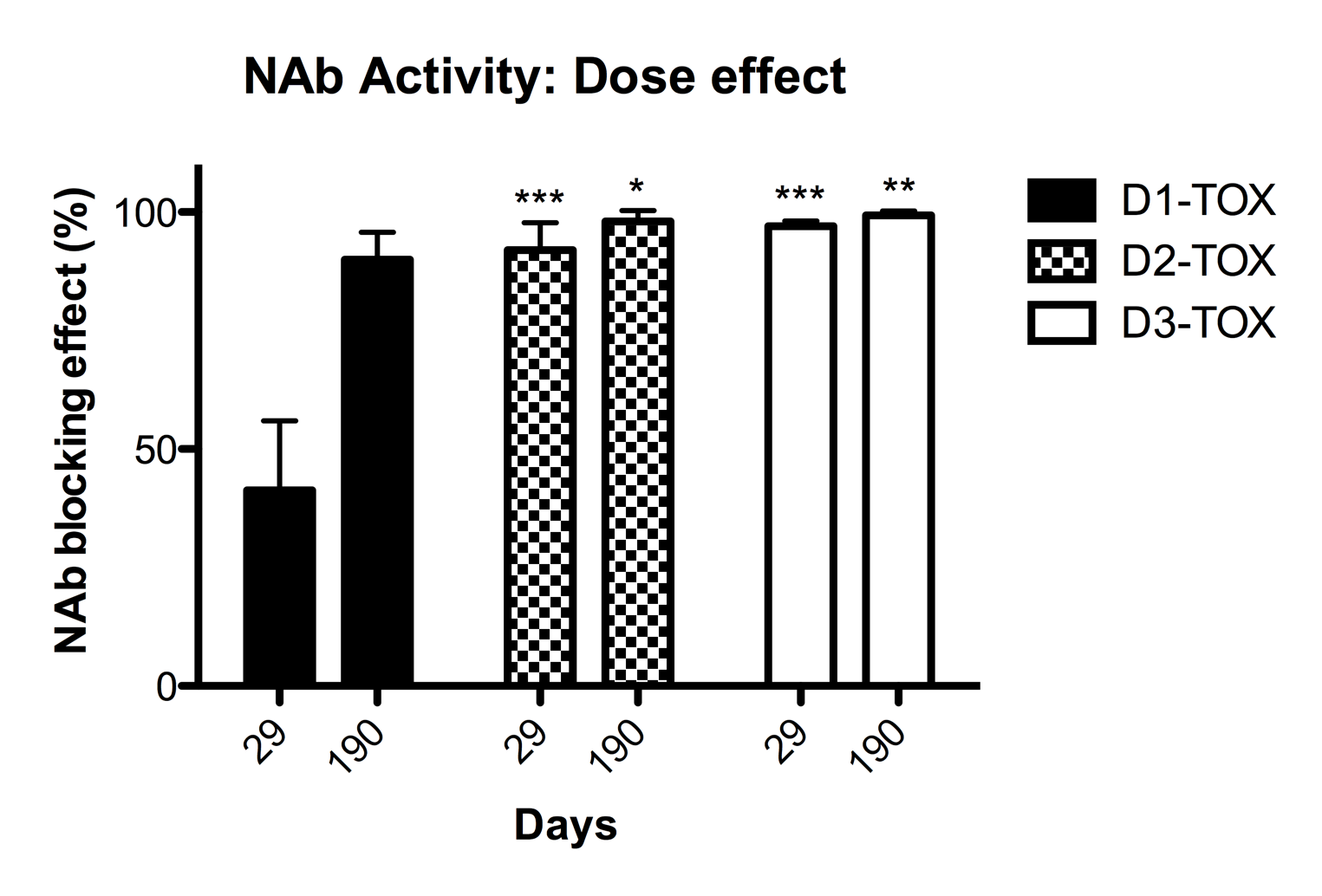

Supplement: S2 Fig — Bars present NAb blocking effect ± SEM. Statistical analyses has been performed with LSD post hoc comparisons test. (TIFF) [file pone.0182715.s009.tiff]

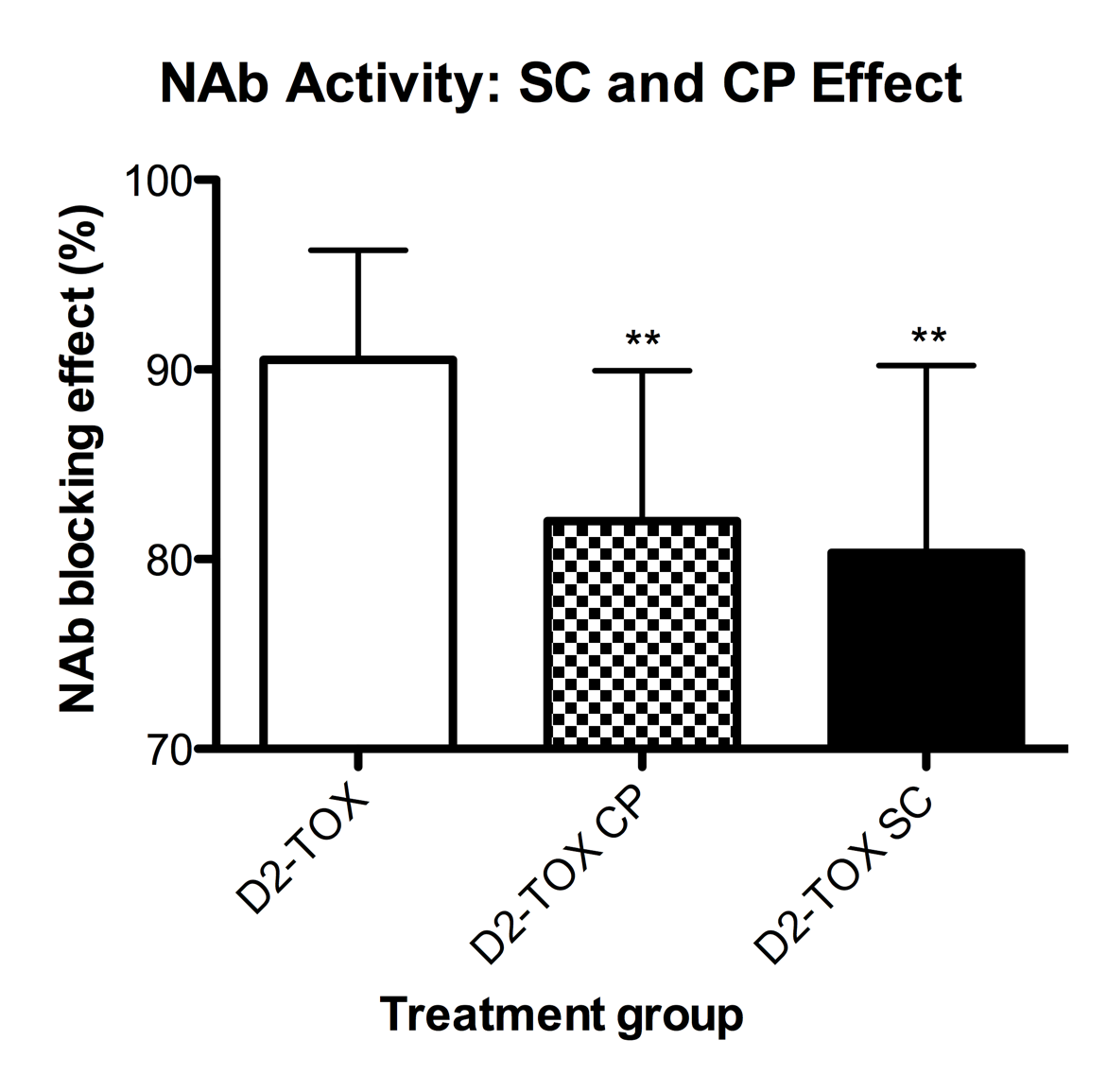

Supplement: S3 Fig — Bars present NAb blocking effect ± SEM. Statistical analyses has been performed with LSD post hoc comparisons test (*** = P≤ 0.001; ** = P≤ 0.01 and * = P≤ 0.05). (TIFF) [file pone.0182715.s010.tiff]

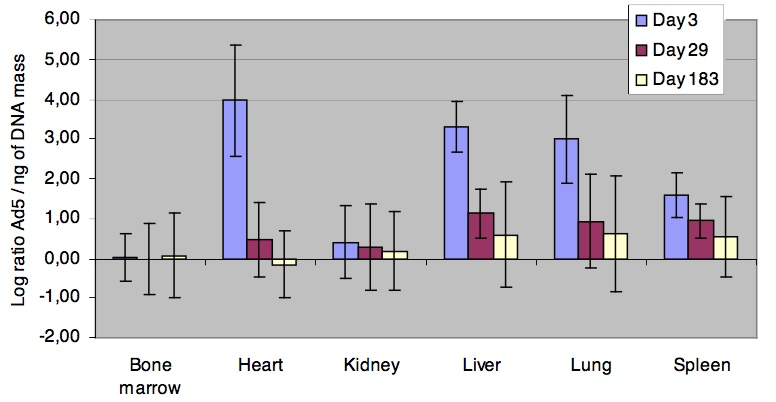

Supplement: S4 Fig — Brain, Gonads, Optic nerves, Feces, Buccal swabs and Urine are not plotted. The whiskers represent standard deviation. (TIF) [file pone.0182715.s011.tif]
